# Supplementary material for: Comparison between gradients and parcellations for functional connectivity prediction of behavior
Source: Neuroimage. Author manuscript; Available in PMC 2023 Jun 1. (PMC10192836; doi:10.1016/j.neuroimage.2023.120044)
Supplement: 1 [file NIHMS1896481-supplement-1.docx]

# Supplementary Figures

Table S1. Site clusters for ABCD

| **ABCD Site** | **Make** | **Model** | **N** | **Site-cluster** |
| --- | --- | --- | --- | --- |
| 16 | Siemens | Prisma | 187 | A |
| 13 | GE | Discovery MR750 | 164 | B |
| 4 | GE | Discovery MR750 | 150 | C |
| 22 | GE | Discovery MR750 | 11 | C |
| 14 | Siemens | Prisma/Prisma fit | 121 | D |
| 15 | Siemens | Prisma fit | 23 | D |
| 10 | GE | Discovery MR750 | 120 | E |
| 11 | Siemens | Prisma | 44 | E |
| 3 | Siemens | Prisma | 111 | F |
| 5 | Siemens | Prisma fit | 61 | F |
| 2 | Siemens | Prisma fit | 68 | G |
| 7 | Siemens | Prisma fit | 56 | G |
| 6 | Siemens | Prisma fit | 47 | H |
| 8 | GE | Discovery MR750 | 55 | H |
| 9 | Siemens | Prisma fit | 20 | H |
| 20 | Siemens | Prisma/Prisma fit | 61 | H |
| 12 | Siemens | Prisma fit | 50 | I |
| 18 | GE | Discovery MR750 | 72 | I |
| 21 | Siemens | Prisma fit/Prisma | 55 | I |


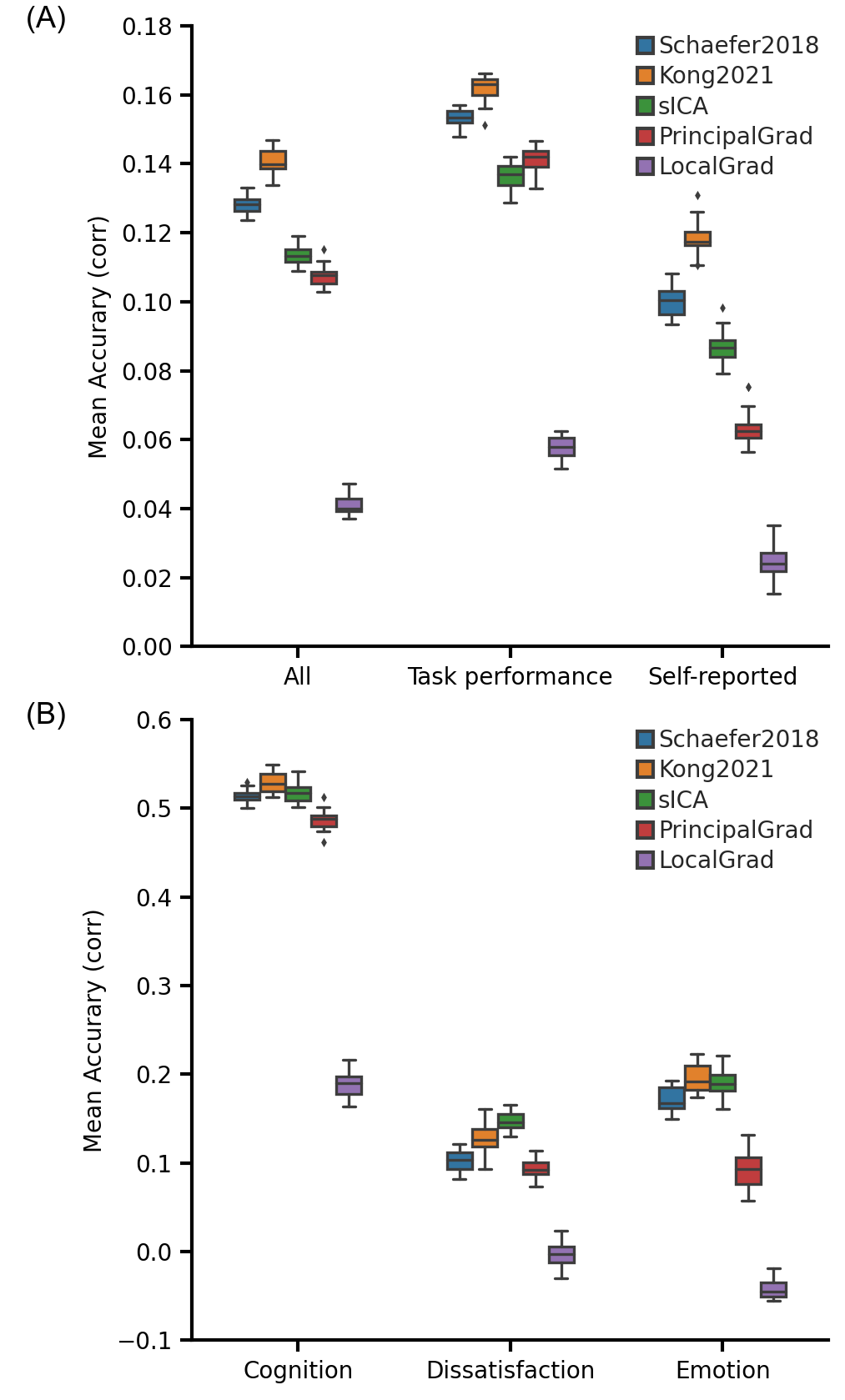


Figure S1. Individual-specific hard-parcellation approach Kong2021 compared favorably with other approaches for linear ridge regression (LRR) in the HCP dataset. (A) Average prediction accuracies (Pearson’s correlation) of all 58 behavioral measures, task performance measures, and self-reported measures. (B) Prediction accuracies (Pearson’s correlation) of three behavioral components: cognition, dissatisfaction, and emotion. Boxplots utilized default Python seaborn parameters, that is, box shows median and interquartile range (IQR). Whiskers indicate 1.5 IQR.


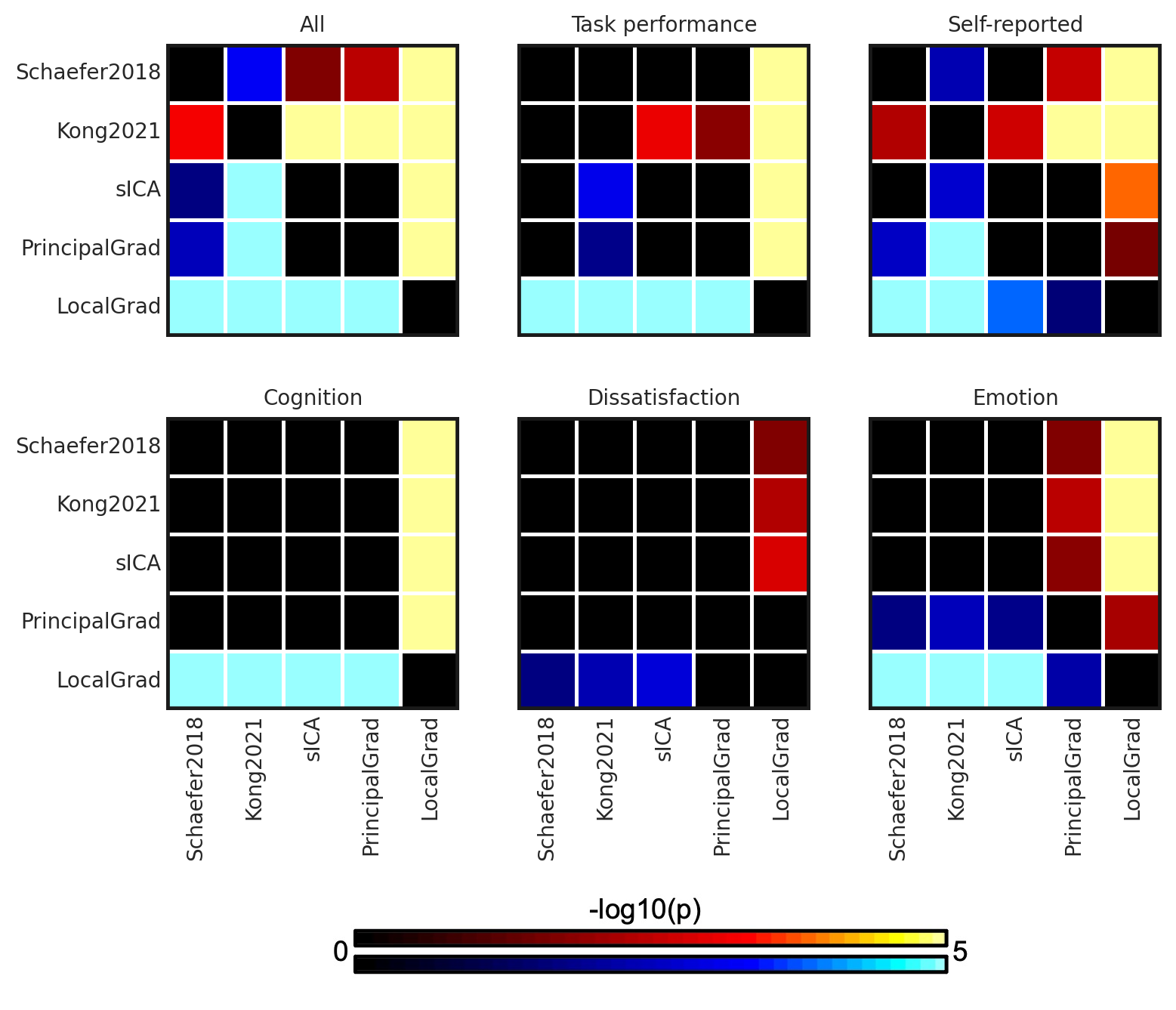


Figure S2. P values (-log10(p)) of comparing prediction accuracies between each pair of approaches for linear ridge regression (LRR) in the HCP dataset. Non-black colors denote significantly different prediction performances after correcting for multiple comparisons with FDR q < 0.05. Bright colors indicate small p values, dark colors indicate large p values. For each pair of comparisons, warm colors represent higher prediction accuracies of the “row” approach than the “column” approach. Individual-specific hard-parcellation approach Kong2021 compared favorably with the other approaches, as can be seen from warm colors along the rows corresponding to Kong2021.


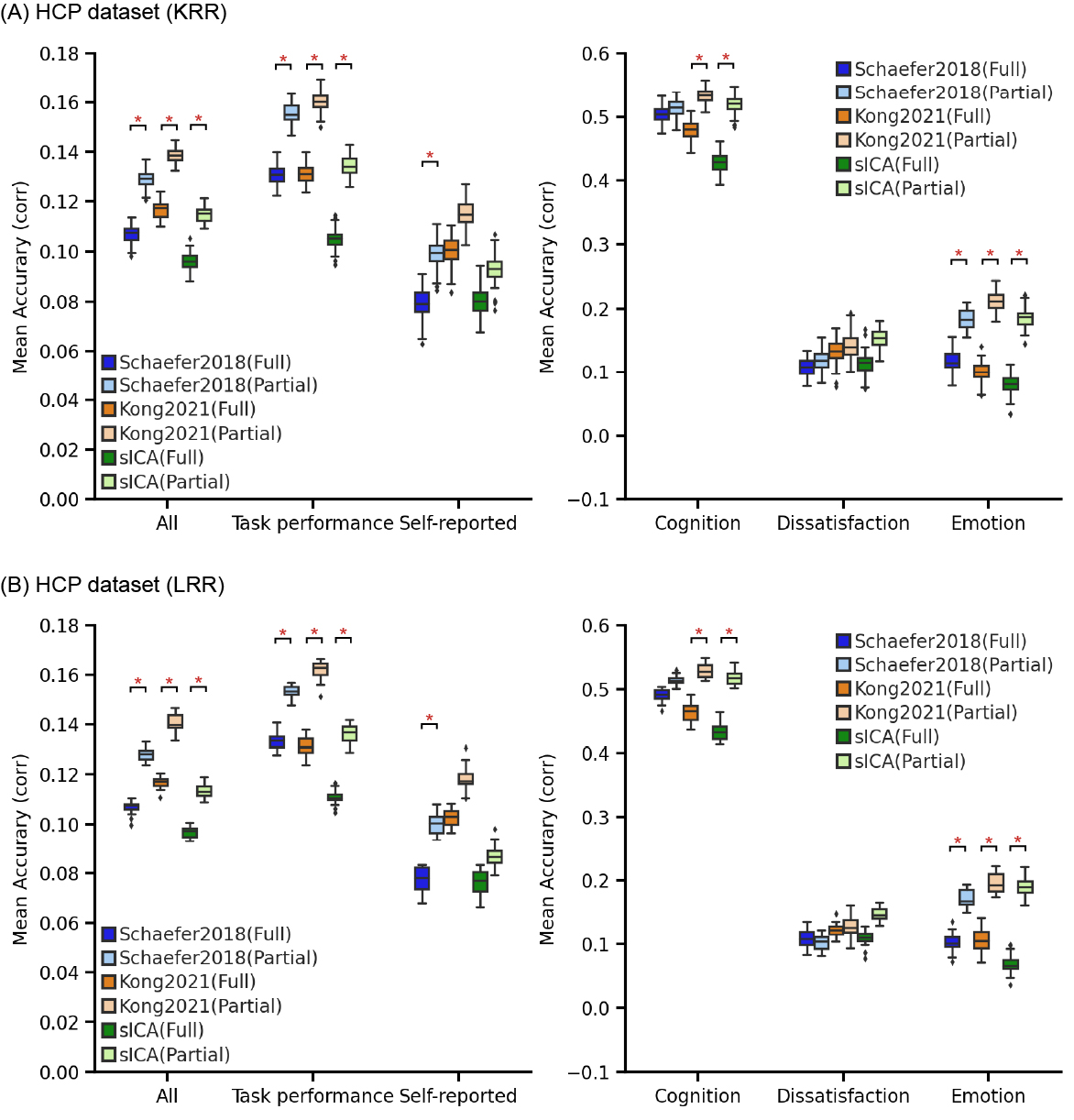


Figure S3. Schaefer2018, Kong201, and sICA using full correlation RSFC performed worse than using partial correlation RSFC in the HCP dataset for (A) kernel ridge regression (KRR) and (B) linear ridge regression (LRR). Boxplots utilized default Python seaborn parameters, that is, box shows median and interquartile range (IQR). Whiskers indicate 1.5 IQR. Red * represents significant p-value after FDR (q < 0.05) correction.


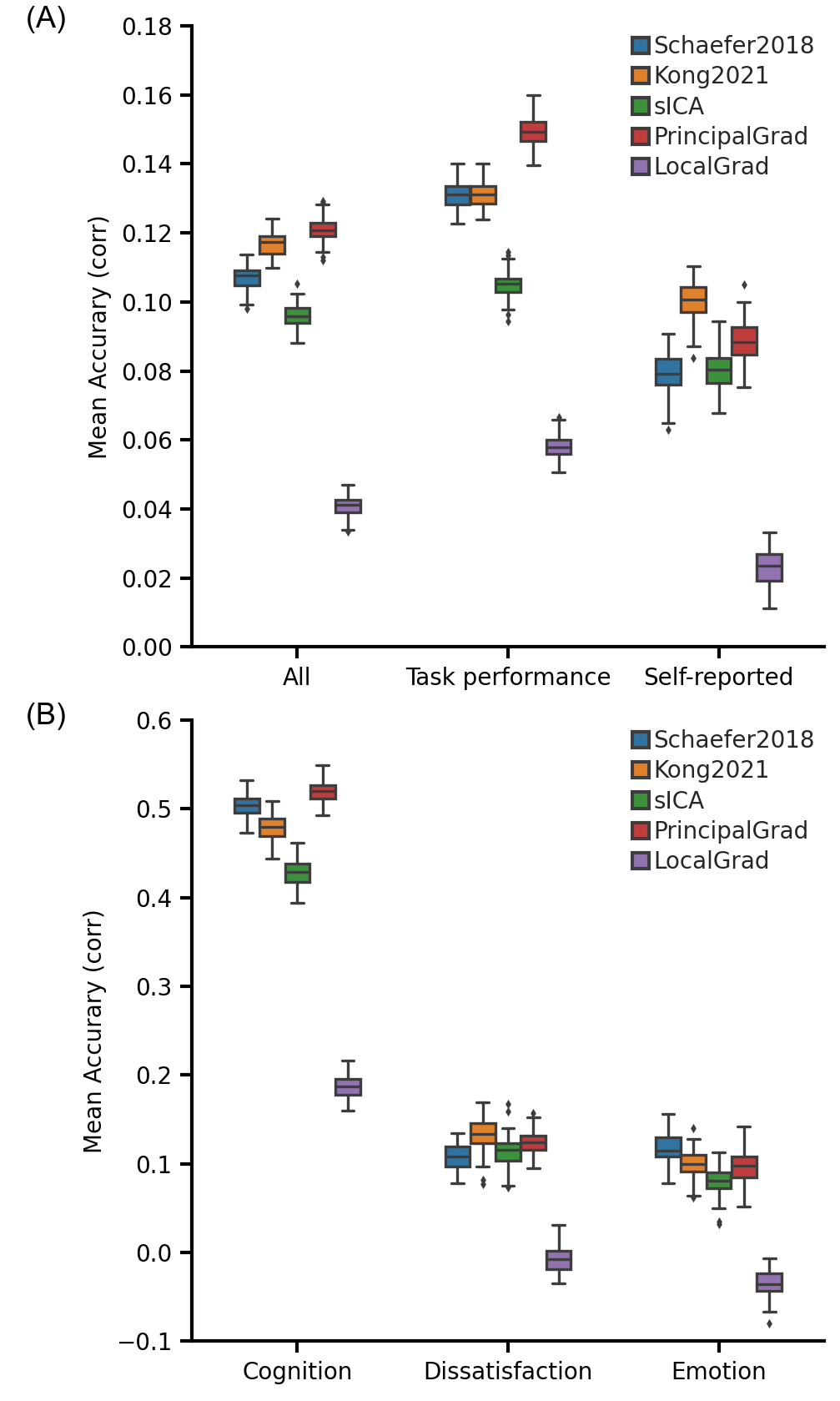


Figure S4. Prediction performance across different approaches using full correlation RSFC for Schaefer2018, Kong2021, and sICA with kernel ridge regression (KRR) in the HCP dataset. (A) Average prediction accuracies (Pearson’s correlation) of all 58 behavioral measures, task performance measures, and self-reported measures. (B) Prediction accuracies (Pearson’s correlation) of three behavioral components: cognition, dissatisfaction, and emotion. Boxplots utilized default Python seaborn parameters, that is, box shows median and interquartile range (IQR). Whiskers indicate 1.5 IQR.


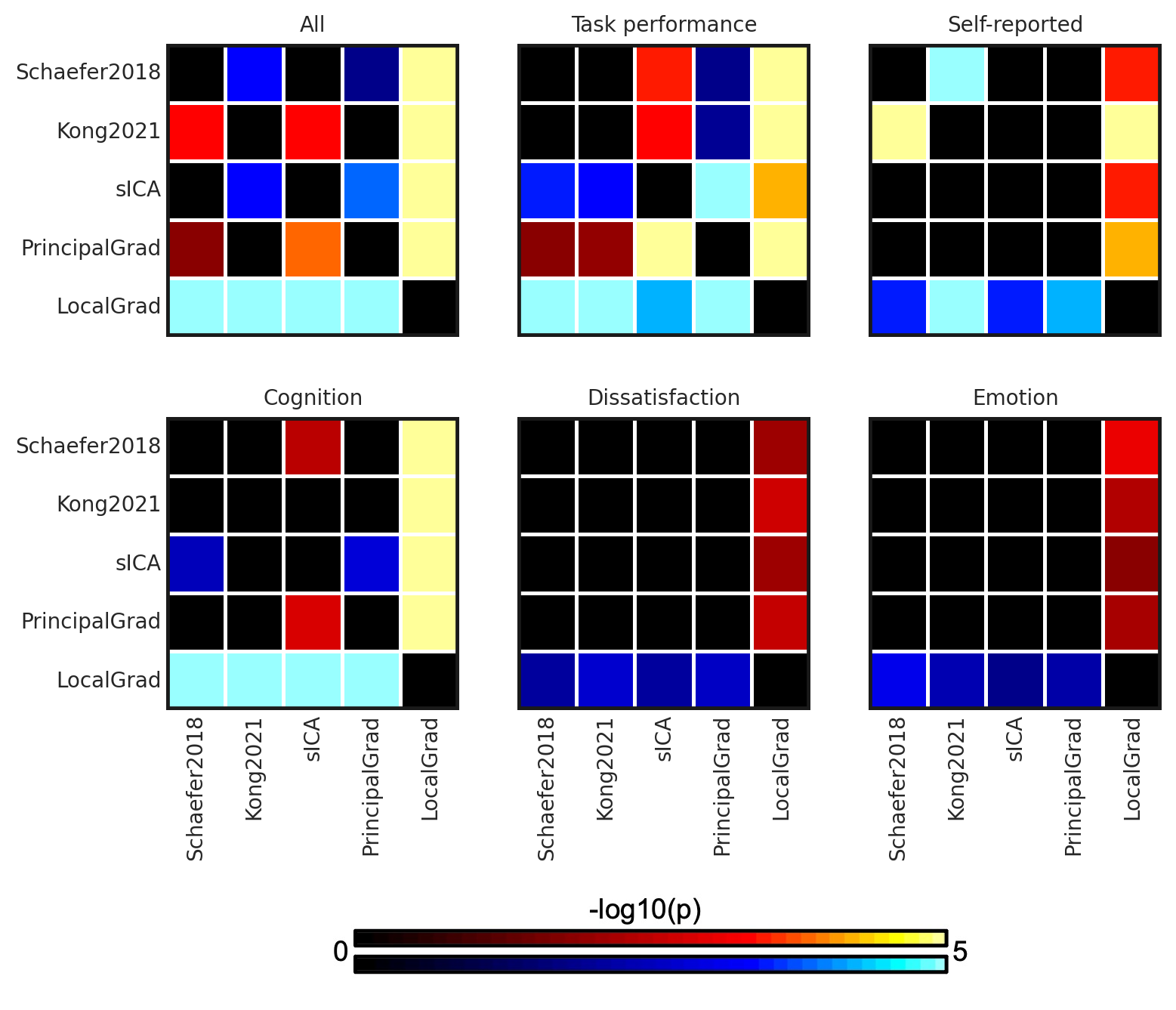


Figure S5. P values (-log10(p)) of comparing prediction accuracies between each pair of approaches for kernel ridge regression (KRR) in the HCP dataset. RSFC of Schaefer2018, Kong2021, and sICA were generated by full correlation. Non-black colors denote significantly different prediction performances after correcting for multiple comparisons with FDR q < 0.05. Bright colors indicate small p values, dark colors indicate large p values. For each pair of comparisons, warm colors represent higher prediction accuracies of the “row” approach than the “column” approach.


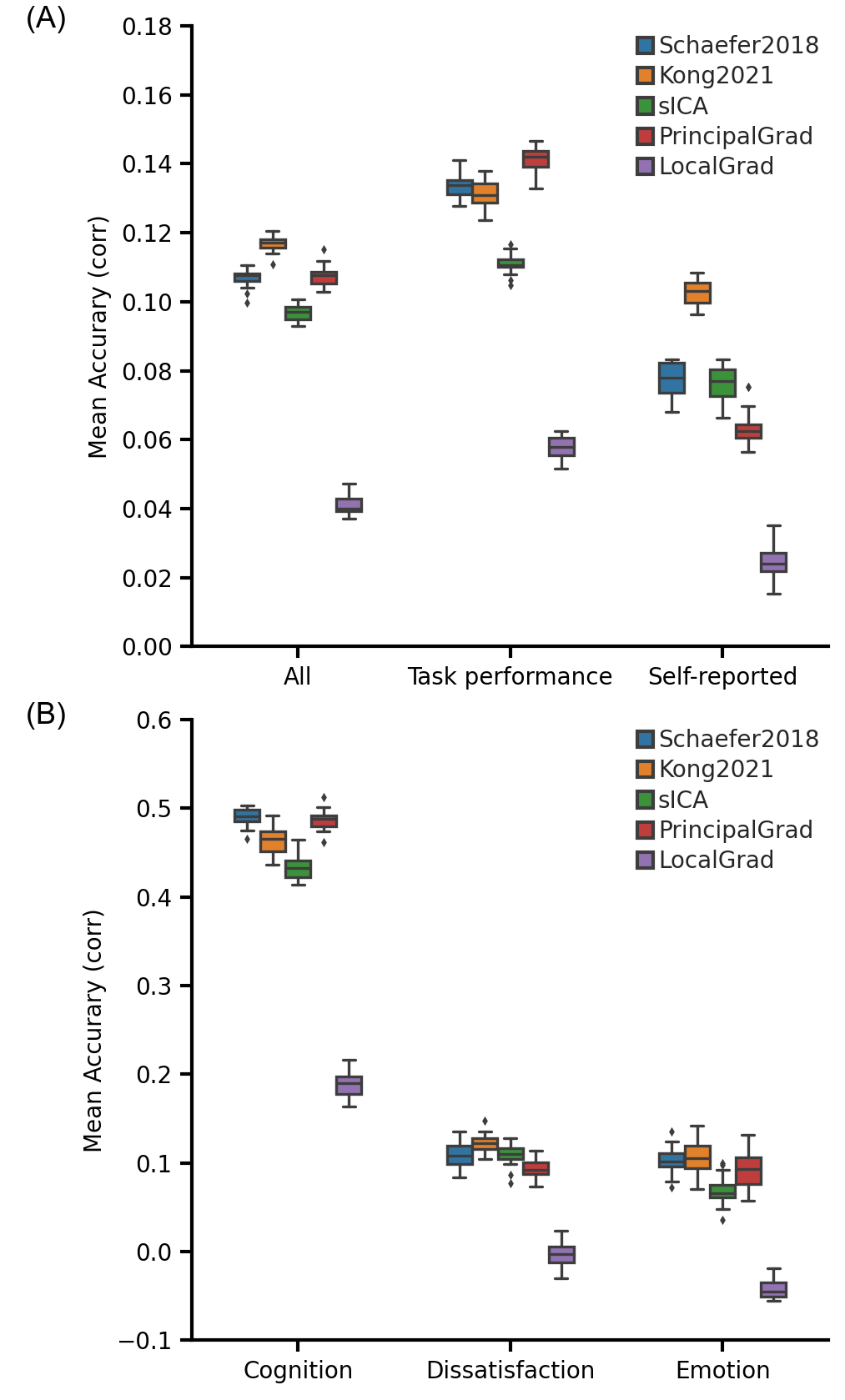


Figure S6. Prediction performance across different approaches using full correlation RSFC for Schaefer2018, Kong2021, and sICA with linear ridge regression (LRR) in the HCP dataset. (A) Average prediction accuracies (Pearson’s correlation) of all 58 behavioral measures, task performance measures, and self-reported measures. (B) Prediction accuracies (Pearson’s correlation) of three behavioral components: cognition, dissatisfaction, and emotion. Boxplots utilized default Python seaborn parameters, that is, box shows median and interquartile range (IQR). Whiskers indicate 1.5 IQR.


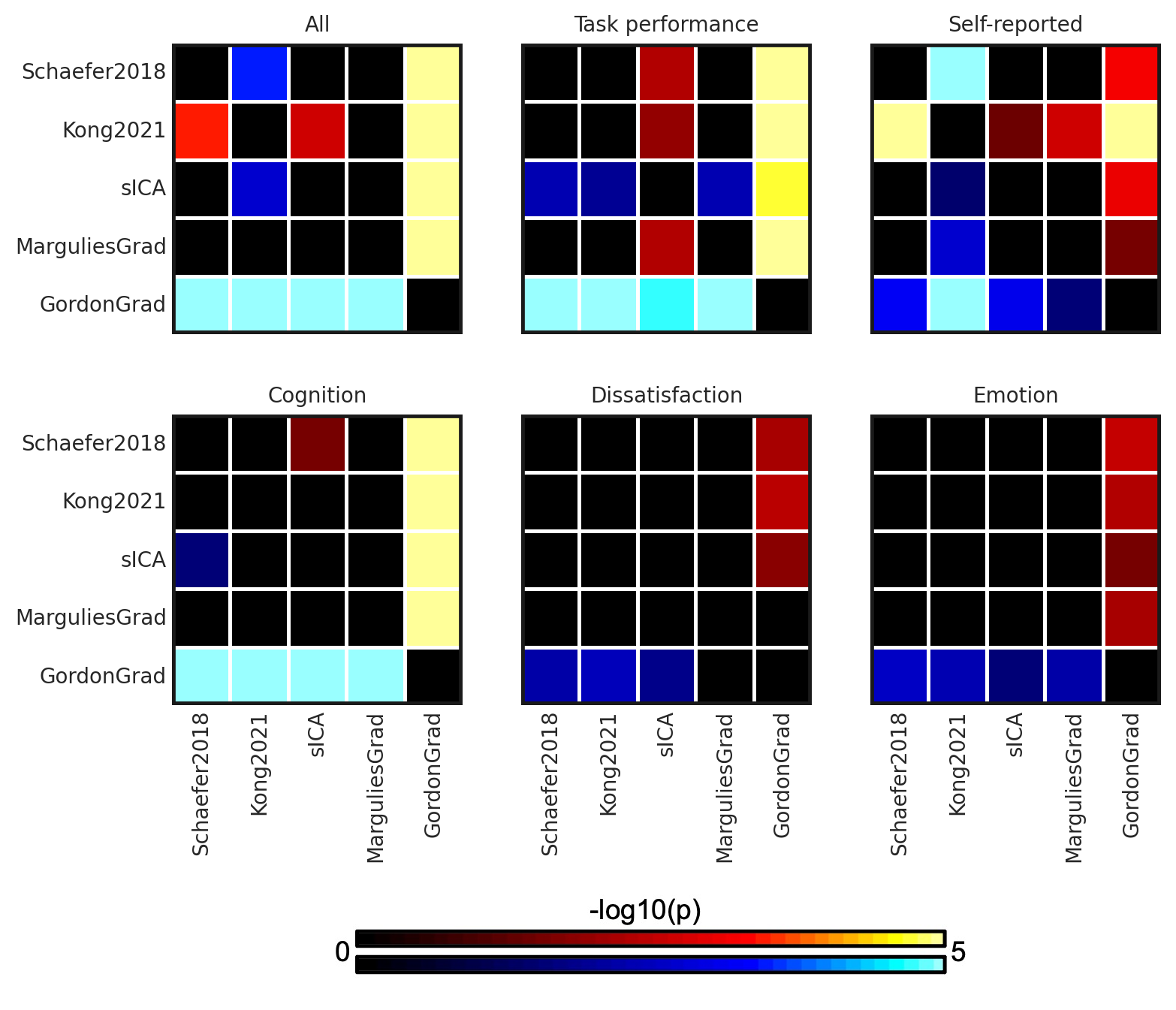


Figure S7. P values (-log10(p)) of comparing prediction accuracies between each pair of approaches for linear ridge regression (LRR) in the HCP dataset. RSFC of Schaefer2018, Kong2021, and sICA were generated by full correlation. Non-black colors denote significantly different prediction performances after correcting for multiple comparisons with FDR q < 0.05. Bright colors indicate small p values, dark colors indicate large p values. For each pair of comparisons, warm colors represent higher prediction accuracies of the “row” approach than the “column” approach.


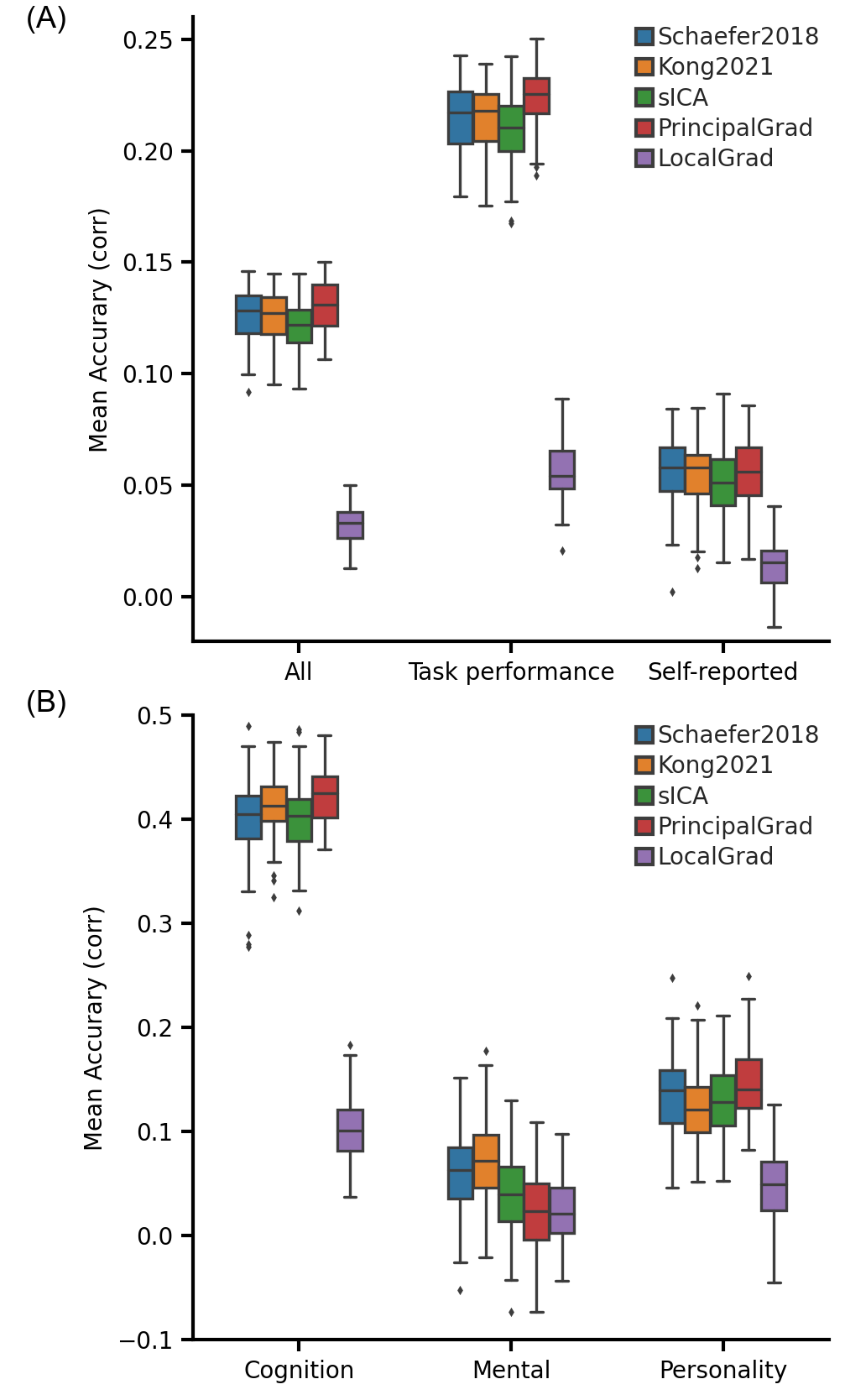


Figure S8. Principal gradient approach achieves comparable behavioral prediction performance as parcellation approaches for linear ridge regression (LRR) in the ABCD dataset. (A) Average prediction accuracies (Pearson’s correlation) of all 36 behavioral measures, task performance measures, and self-reported measures. (B) Prediction accuracies (Pearson’s correlation) of three behavioral components: cognition, mental health, and personality. Boxplots utilized default Python seaborn parameters, that is, box shows median and interquartile range (IQR). Whiskers indicate 1.5 IQR. The principal gradient approach PrincipalGrad was numerically the best for most cases, but there was largely no statistical difference among the approaches.


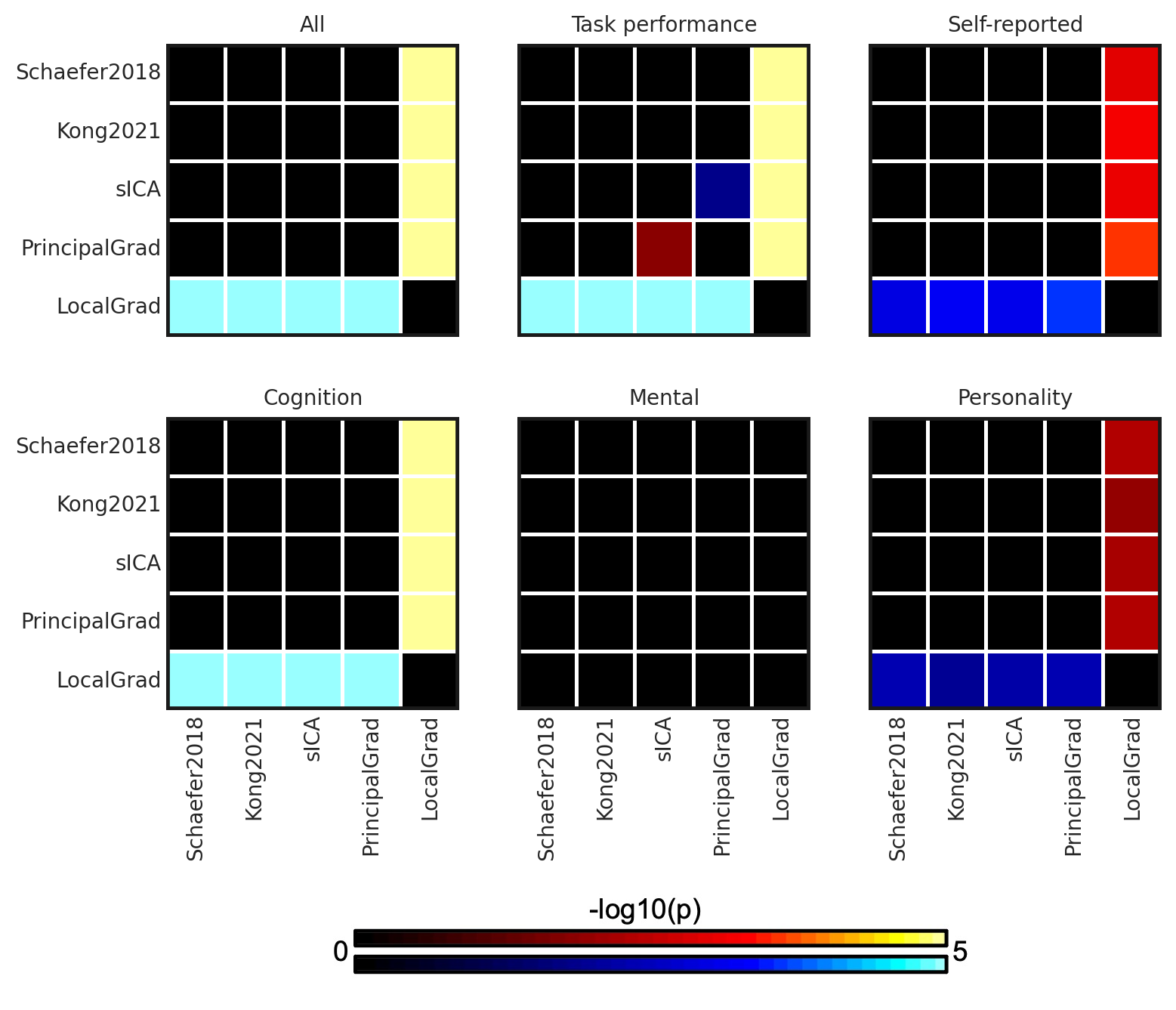


Figure S9. P values (-log10(p)) of comparing prediction accuracies between each pair of approaches for linear ridge regression (LRR) in the ABCD dataset. Non-black colors denote significantly different prediction performances after correcting for multiple comparisons with FDR q < 0.05. Bright colors indicate small p values, dark colors indicate large p values. For each pair of comparisons, warm colors represent higher prediction accuracies of the “row” approach than the “column” approach. There was no statistical difference among most approaches.


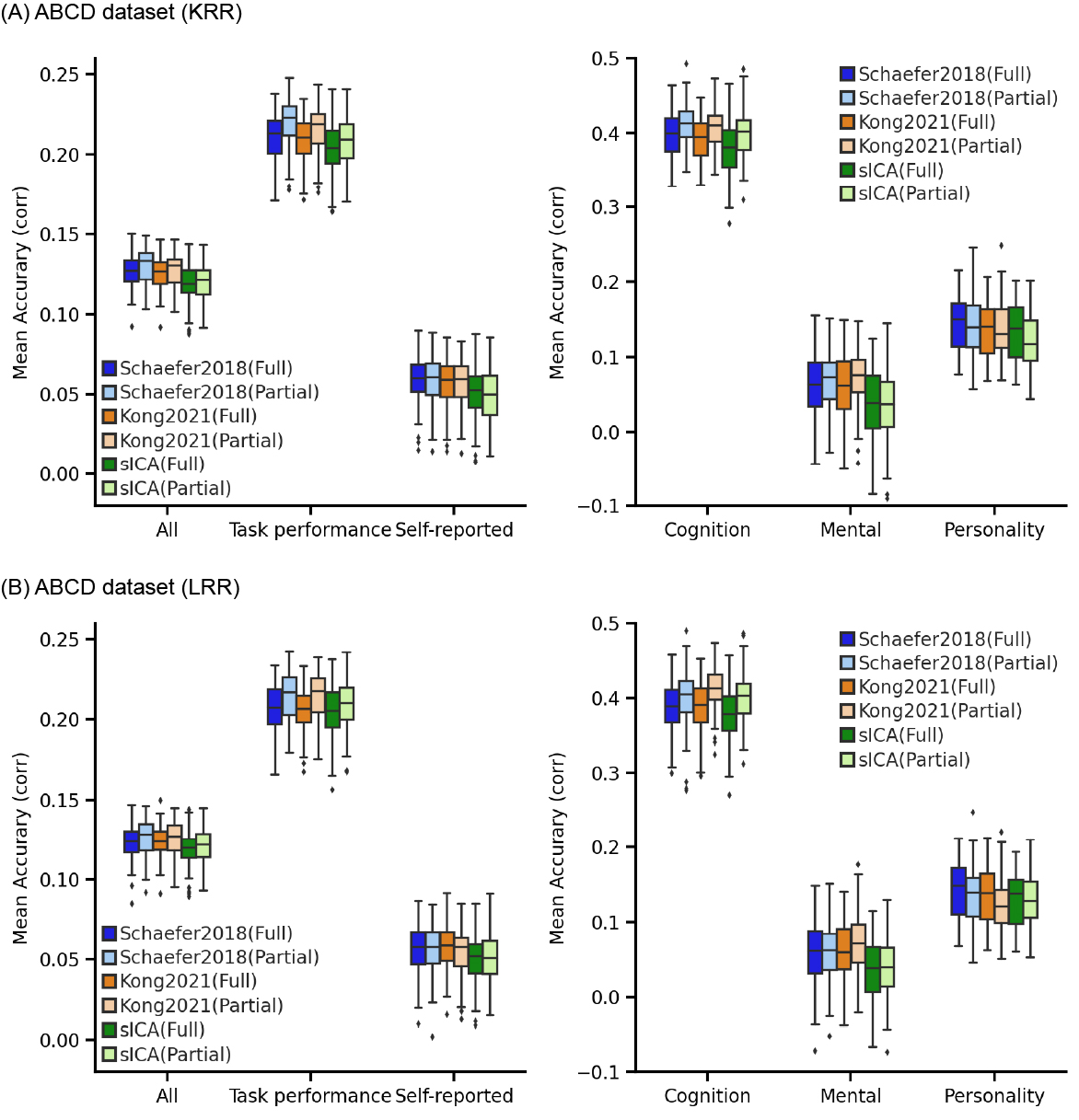


Figure S10. Schaefer2018, Kong201, and sICA using full correlation RSFC performed numerically worse than using partial correlation RSFC in the ABCD dataset for (A) kernel ridge regression (KRR) and (B) linear ridge regression (LRR), but differences were not significant. Boxplots utilized default Python seaborn parameters, that is, box shows median and interquartile range (IQR). Whiskers indicate 1.5 IQR.


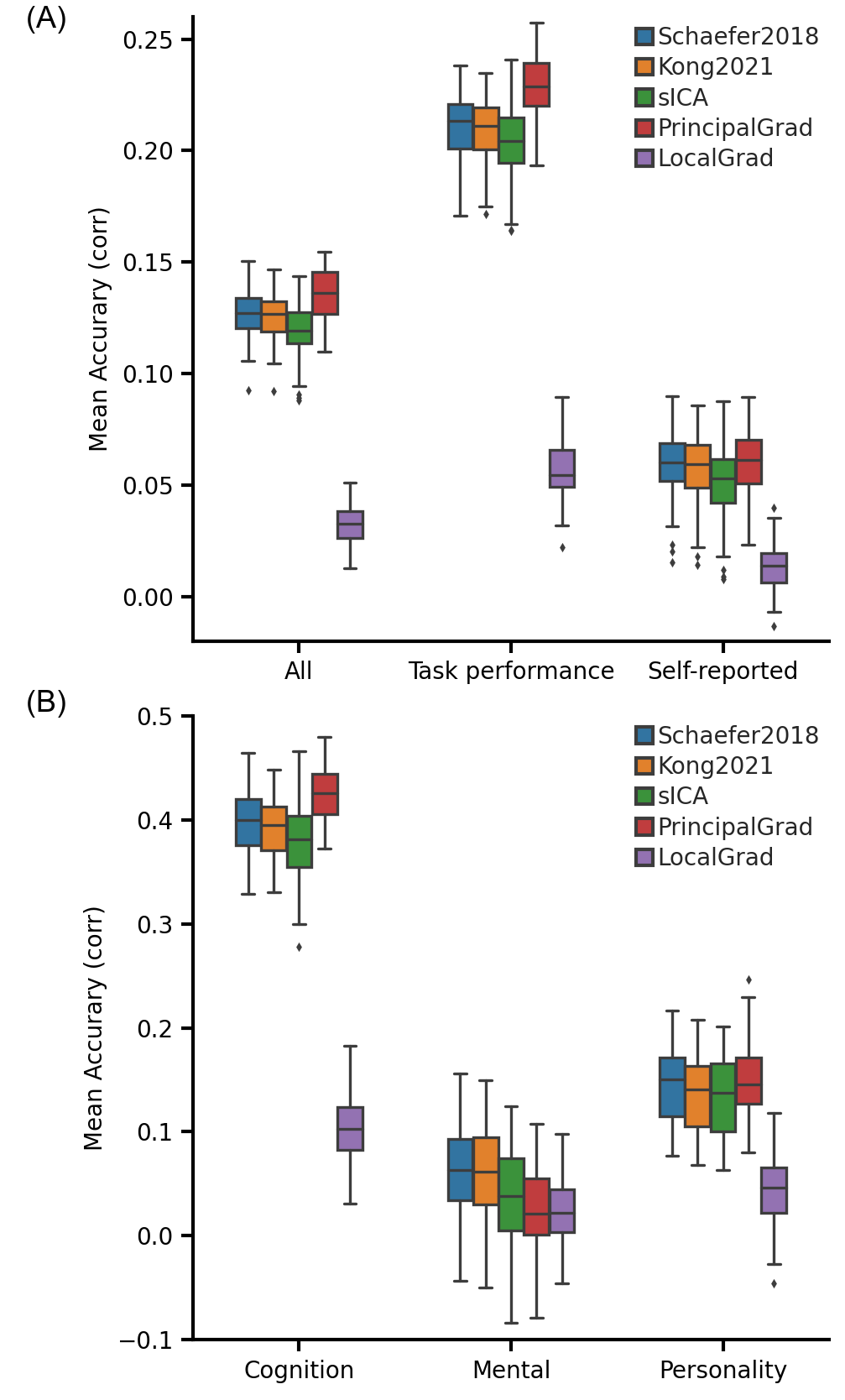


Figure S11. Prediction performance across different approaches using full correlation RSFC for Schaefer2018, Kong2021, and sICA with kernel ridge regression (KRR) in the ABCD dataset. (A) Average prediction accuracies (Pearson’s correlation) of all 36 behavioral measures, task performance measures, and self-reported measures. (B) Prediction accuracies (Pearson’s correlation) of three behavioral components: cognition, mental health, and personality. Boxplots utilized default Python seaborn parameters, that is, box shows median and interquartile range (IQR). Whiskers indicate 1.5 IQR. The principal gradient approach PrincipalGrad was numerically the best for most cases, but there was largely no statistical difference among the approaches.


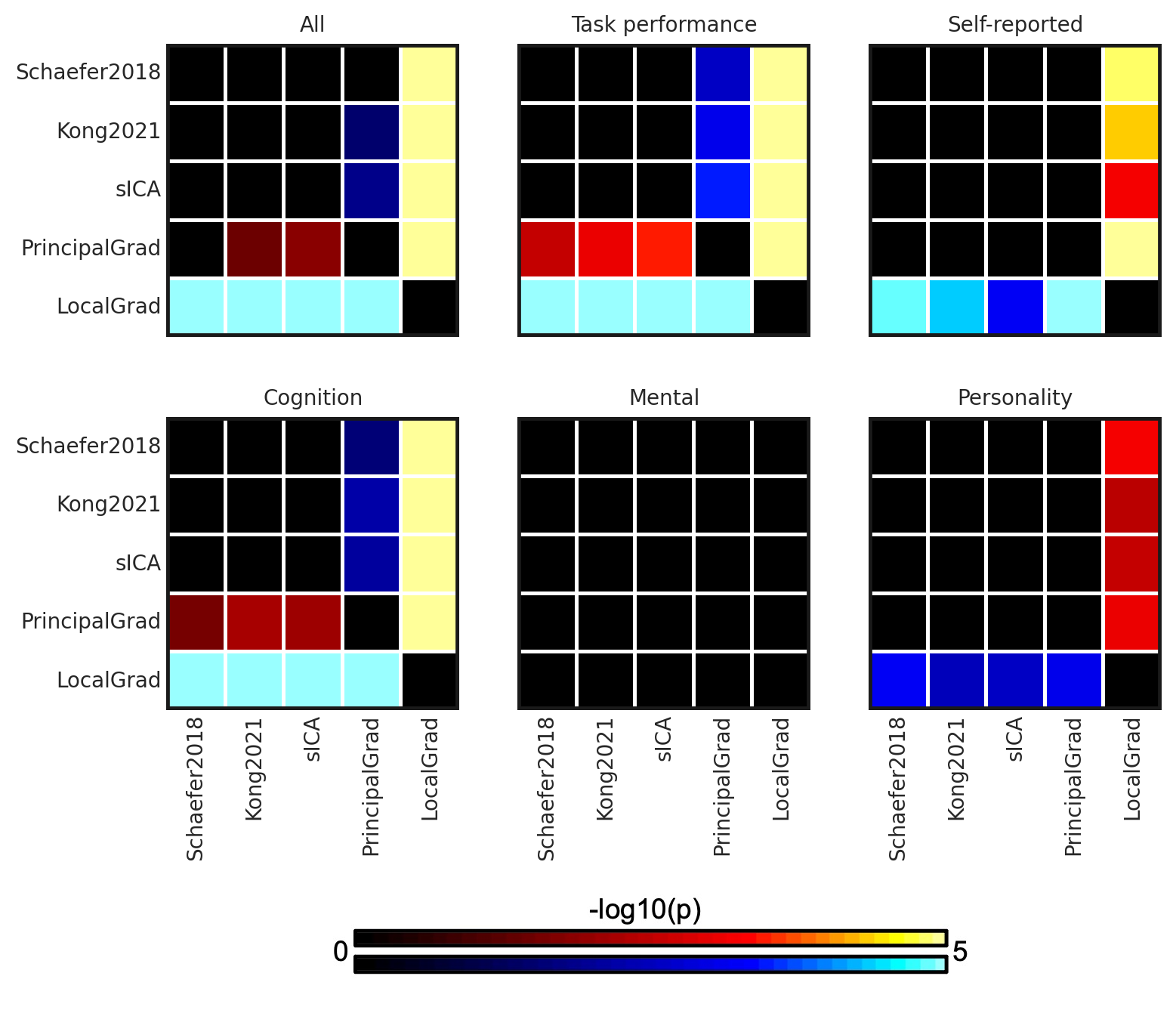


Figure S12. P values (-log10(p)) of comparing prediction accuracies between each pair of approaches for kernel ridge regression (KRR) in the ABCD dataset. RSFC of Schaefer2018, Kong2021, and sICA were generated by full correlation. Non-black colors denote significantly different prediction performances after correcting for multiple comparisons with FDR q < 0.05. Bright colors indicate small p values, dark colors indicate large p values. For each pair of comparisons, warm colors represent higher prediction accuracies of the “row” approach than the “column” approach.


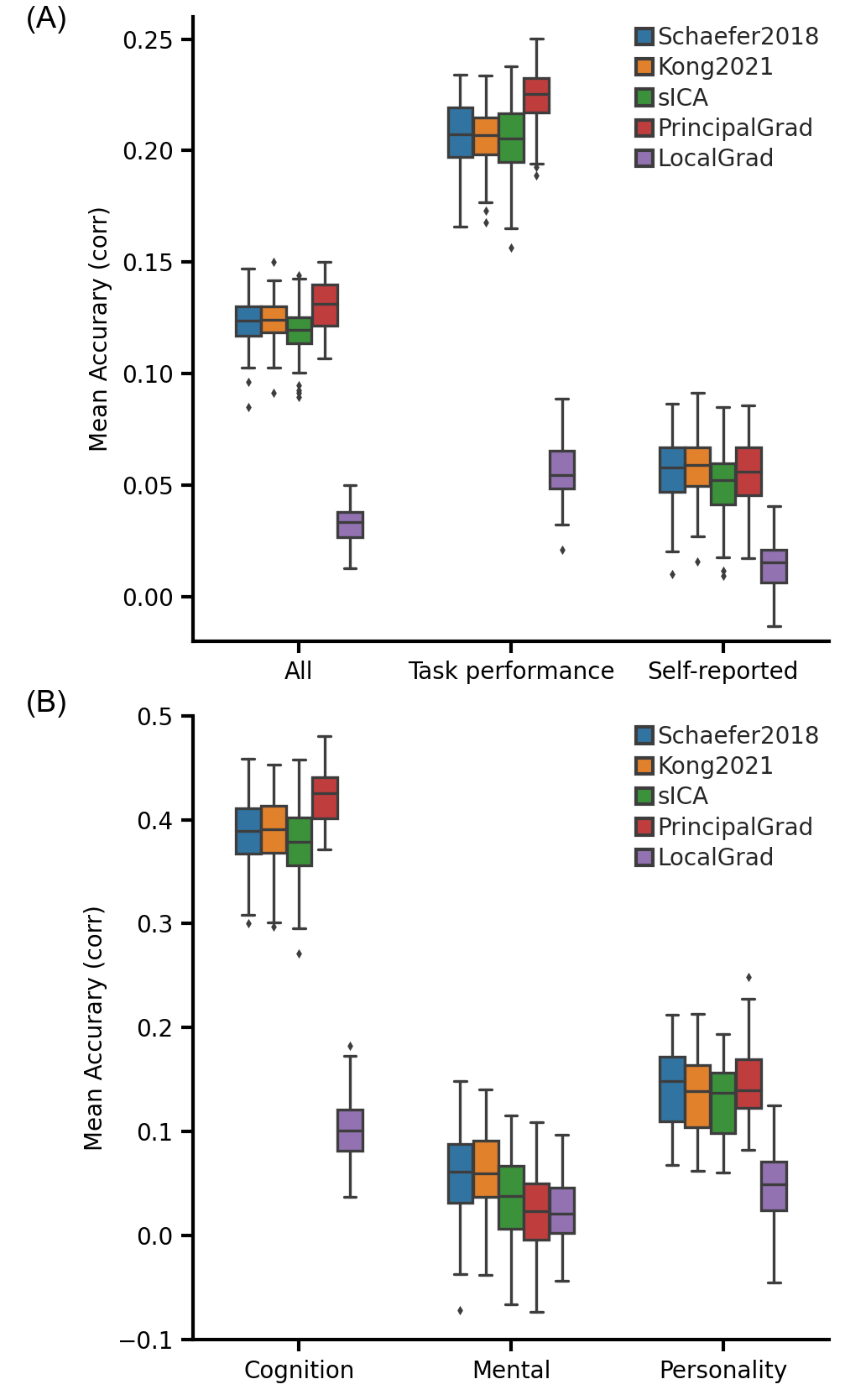


Figure S13. Prediction performance across different approaches using full correlation RSFC for Schaefer2018, Kong2021, and sICA with linear ridge regression (LRR) in the ABCD dataset. (A) Average prediction accuracies (Pearson’s correlation) of all 36 behavioral measures, task performance measures, and self-reported measures. (B) Prediction accuracies (Pearson’s correlation) of three behavioral components: cognition, mental health, and personality. Boxplots utilized default Python seaborn parameters, that is, box shows median and interquartile range (IQR). Whiskers indicate 1.5 IQR.


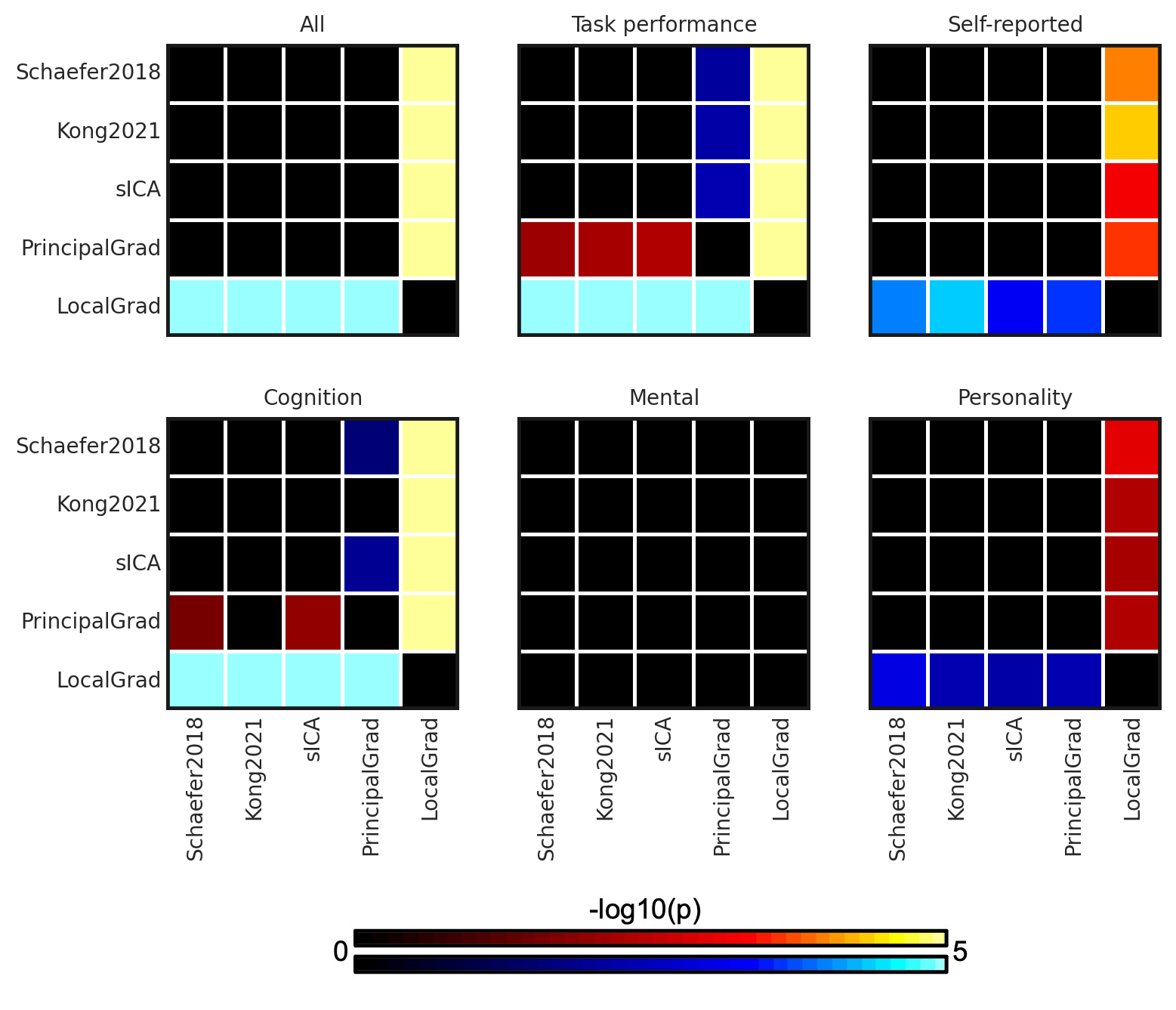


Figure S14. P values (-log10(p)) of comparing prediction accuracies between each pair of approaches for linear ridge regression (LRR) in the ABCD dataset. RSFC of Schaefer2018, Kong2021, and sICA were generated by full correlation. Non-black colors denote significantly different prediction performances after correcting for multiple comparisons with FDR q < 0.05. Bright colors indicate small p values, dark colors indicate large p values. For each pair of comparisons, warm colors represent higher prediction accuracies of the “row” approach than the “column” approach.


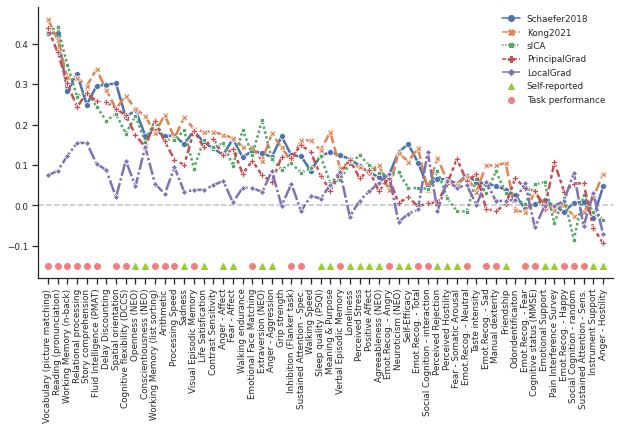


Figure S15. Task performance measures were predicted better than self-reported measures across different gradient and parcellation approaches with optimized resolutions for LRR in the HCP dataset. 58 behavioral measures were ordered based on average prediction accuracies across Schaefer2018, Kong2021, sICA, PrincipalGrad, and LocalGrad. Pink circles indicate task performance measures. Green triangles indicate self-reported measures. There were more task performance measures (pink circles) on the left side of x-axis. Boxplots utilized default Python seaborn parameters, that is, box shows median and interquartile range (IQR). Whiskers indicate 1.5 IQR. Designation of behavioral measures into “self-reported” and “task-performance” measures followed previous studies (Li et al., 2019a; Liégeois et al., 2019; Kong et al., 2021a).


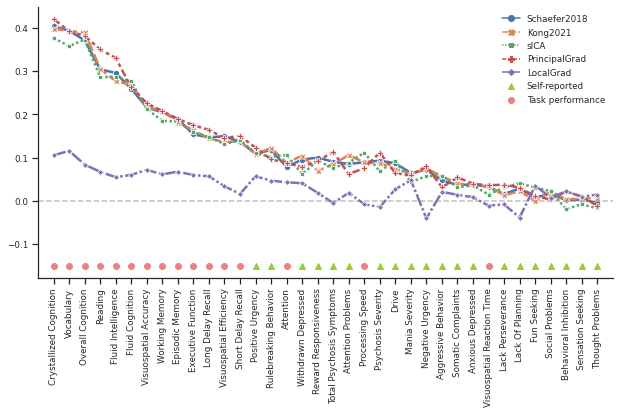


Figure S16. Task performance measures were predicted better than self-reported measures across different gradient and parcellation approaches with optimized resolutions for LRR in the ABCD dataset. 36 behavioral measures were ordered based on average prediction accuracies across Schaefer2018, Kong2021, sICA, PrincipalGrad, and LocalGrad. Pink circles indicate task performance measures. Green triangles indicate self-reported measures. There were more task performance measures (pink circles) on the left side of x-axis. Boxplots utilized default Python seaborn parameters, that is, box shows median and interquartile range (IQR). Whiskers indicate 1.5 IQR. Designation of behavioral measures into “self-reported” and “task-performance” measures based on ABCD behavioral measures description (Li et al., 2019a; Liégeois et al., 2019; Kong et al., 2021a).


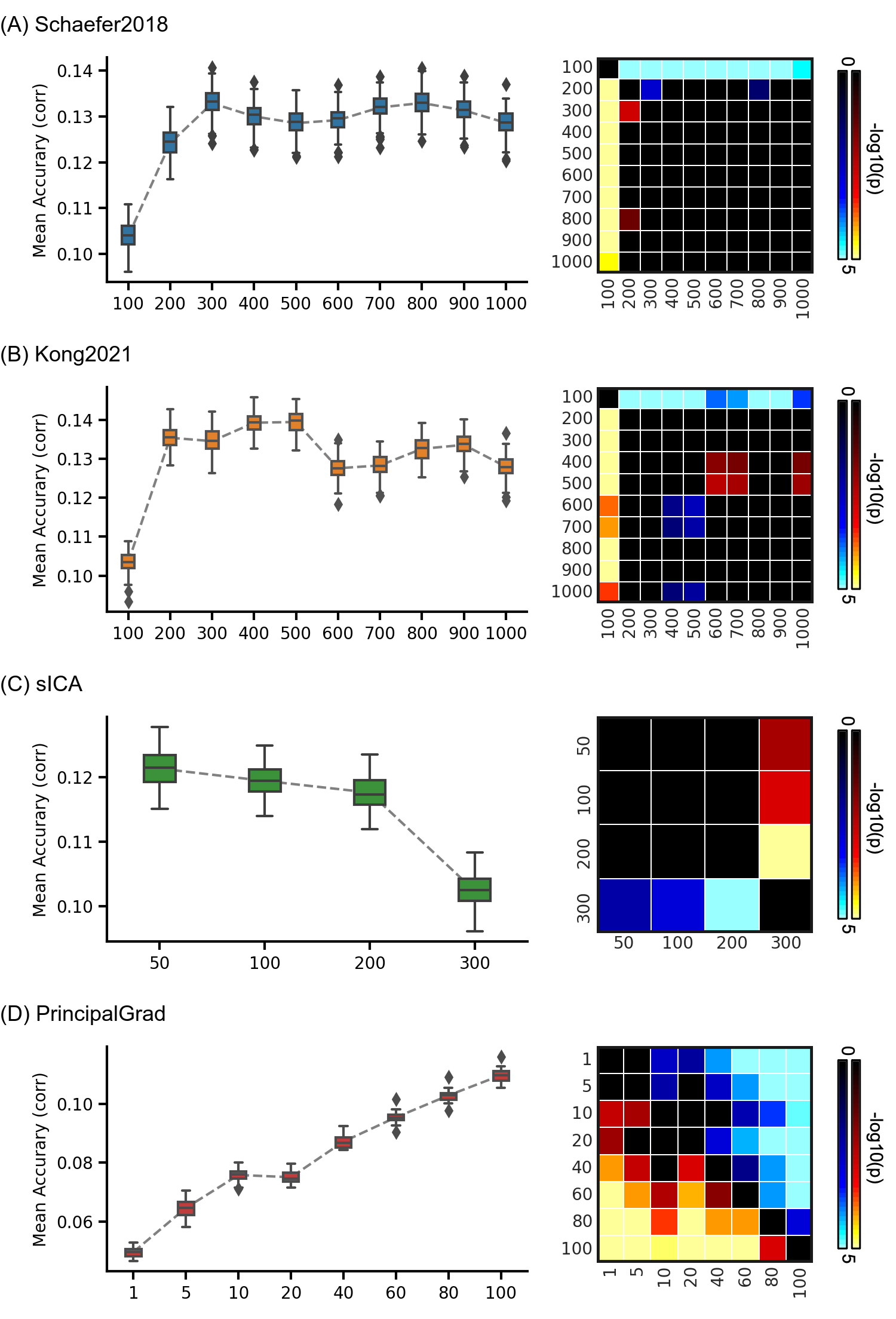


Figure S17. Average prediction accuracies (Pearson’s correlation) of all 58 behavioral measures vary across resolutions for gradient and parcellation approaches using LRR in the HCP dataset. (A) Prediction accuracies and p values of the hard-parcellation Schaefer2018 with 100 to 1000 ROIs. (B) Prediction accuracies and p values of the hard-parcellation Kong2021 with 100 to 1000 ROIs. (C) Prediction accuracies and p values of the soft-parcellation sICA with 50 to 300 components. (D) Prediction accuracies and p values of the principal gradient PrincipalGrad with 1 to 100 gradients. Boxplots utilized default Python seaborn parameters, that is, box shows median and interquartile range (IQR). Whiskers indicate 1.5 IQR. P values (-log10(p)) were computed between prediction accuracies of each pair of resolutions. Non-black colors denote significantly different prediction performances after correcting for multiple comparisons with FDR q < 0.05. Bright colors indicate small p values, dark colors indicate large p values. For each pair of comparisons, warm colors represent higher prediction accuracies of the “row” resolution than the “column” resolution.


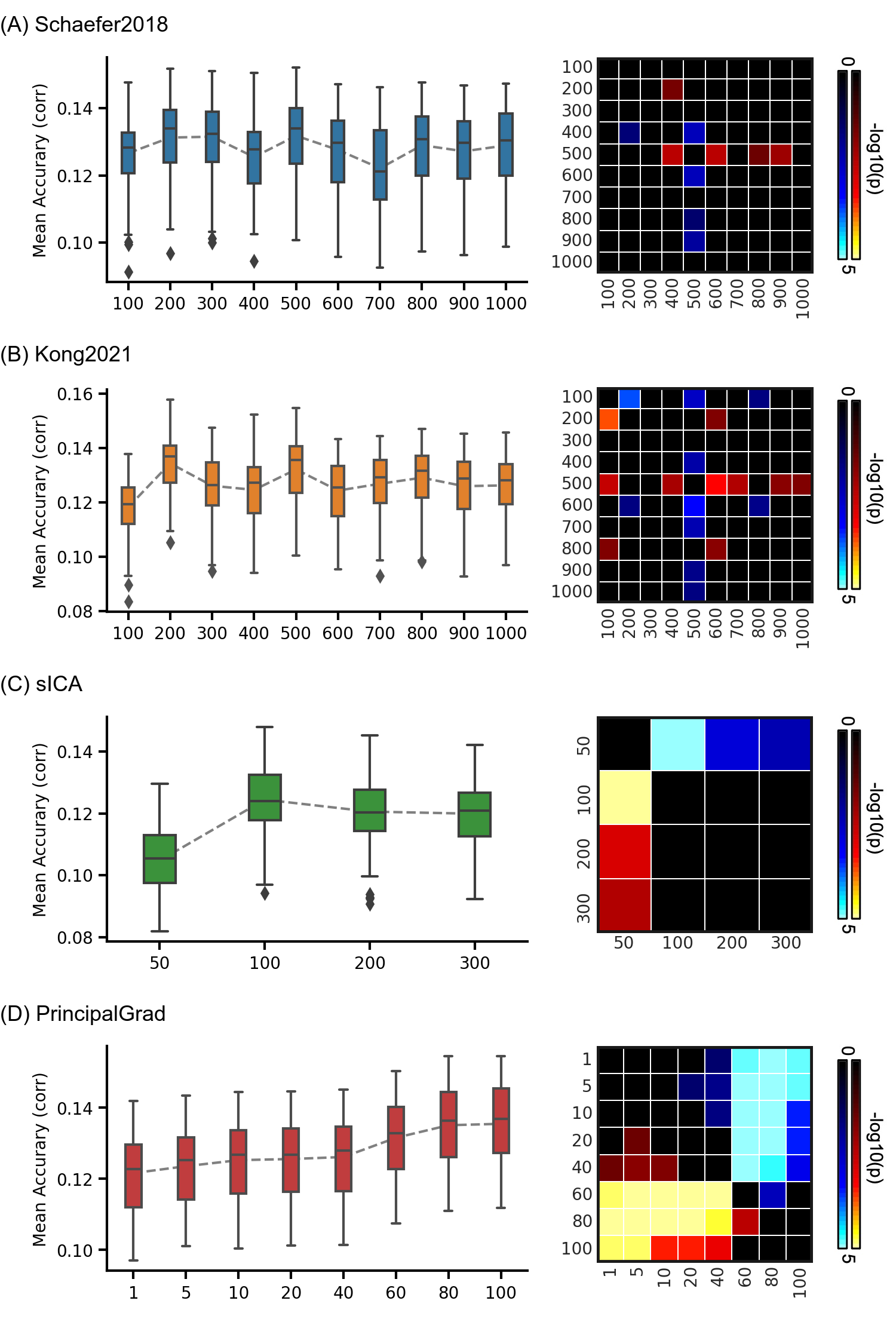


Figure S18. Average prediction accuracies (Pearson’s correlation) of all 36 behavioral measures vary across resolutions for gradient and parcellation approaches using LRR in the ABCD dataset. (A) Prediction accuracies and p values of the hard-parcellation Schaefer2018 with 100 to 1000 ROIs. (B) Prediction accuracies and p values of the hard-parcellation Kong2021 with 100 to 1000 ROIs. (C) Prediction accuracies and p values of the soft-parcellation sICA with 50 to 300 components. (D) Prediction accuracies and p values of the principal gradient PrincipalGrad with 1 to 100 gradients. Boxplots utilized default Python seaborn parameters, that is, box shows median and interquartile range (IQR). Whiskers indicate 1.5 IQR. P values (-log10(p)) were computed between prediction accuracies of each pair of resolutions. Non-black colors denote significantly different prediction performances after correcting for multiple comparisons with FDR q < 0.05. Bright colors indicate small p values, dark colors indicate large p values. For each pair of comparisons, warm colors represent higher prediction accuracies of the “row” resolution than the “column” resolution.


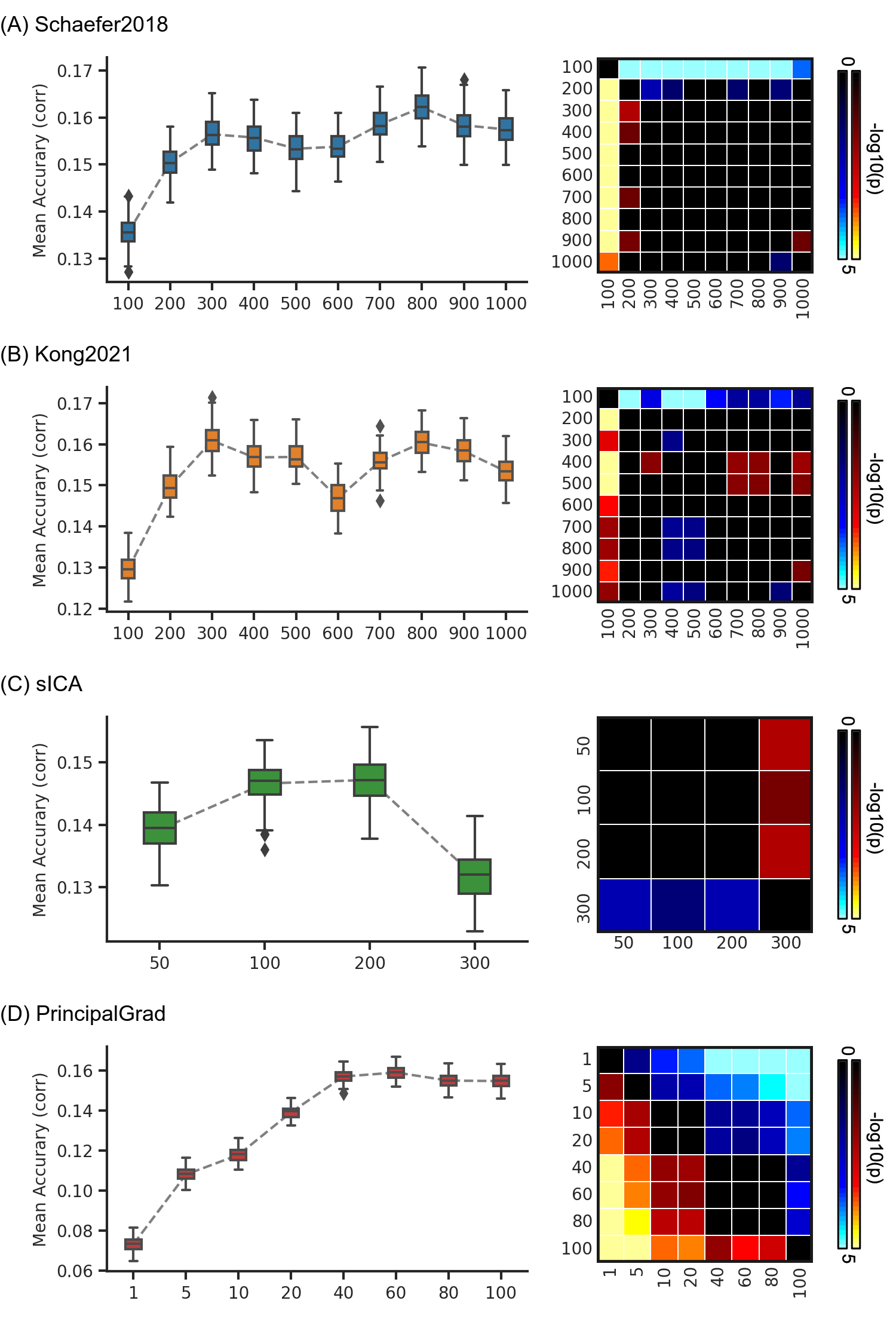


Figure S19. Average prediction accuracies (Pearson’s correlation) of task performance measures vary across resolutions for gradient and parcellation approaches using KRR in the HCP dataset. (A) Prediction accuracies and p values of the hard-parcellation Schaefer2018 with 100 to 1000 ROIs. (B) Prediction accuracies and p values of the hard-parcellation Kong2021 with 100 to 1000 ROIs. (C) Prediction accuracies and p values of the soft-parcellation sICA with 50 to 300 components. (D) Prediction accuracies and p values of the principal gradient PrincipalGrad with 1 to 100 gradients. Boxplots utilized default Python seaborn parameters, that is, box shows median and interquartile range (IQR). Whiskers indicate 1.5 IQR. P values (-log10(p)) were computed between prediction accuracies of each pair of resolutions. Non-black colors denote significantly different prediction performances after correcting for multiple comparisons with FDR q < 0.05. Bright colors indicate small p values, dark colors indicate large p values. For each pair of comparisons, warm colors represent higher prediction accuracies of the “row” resolution than the “column” resolution.


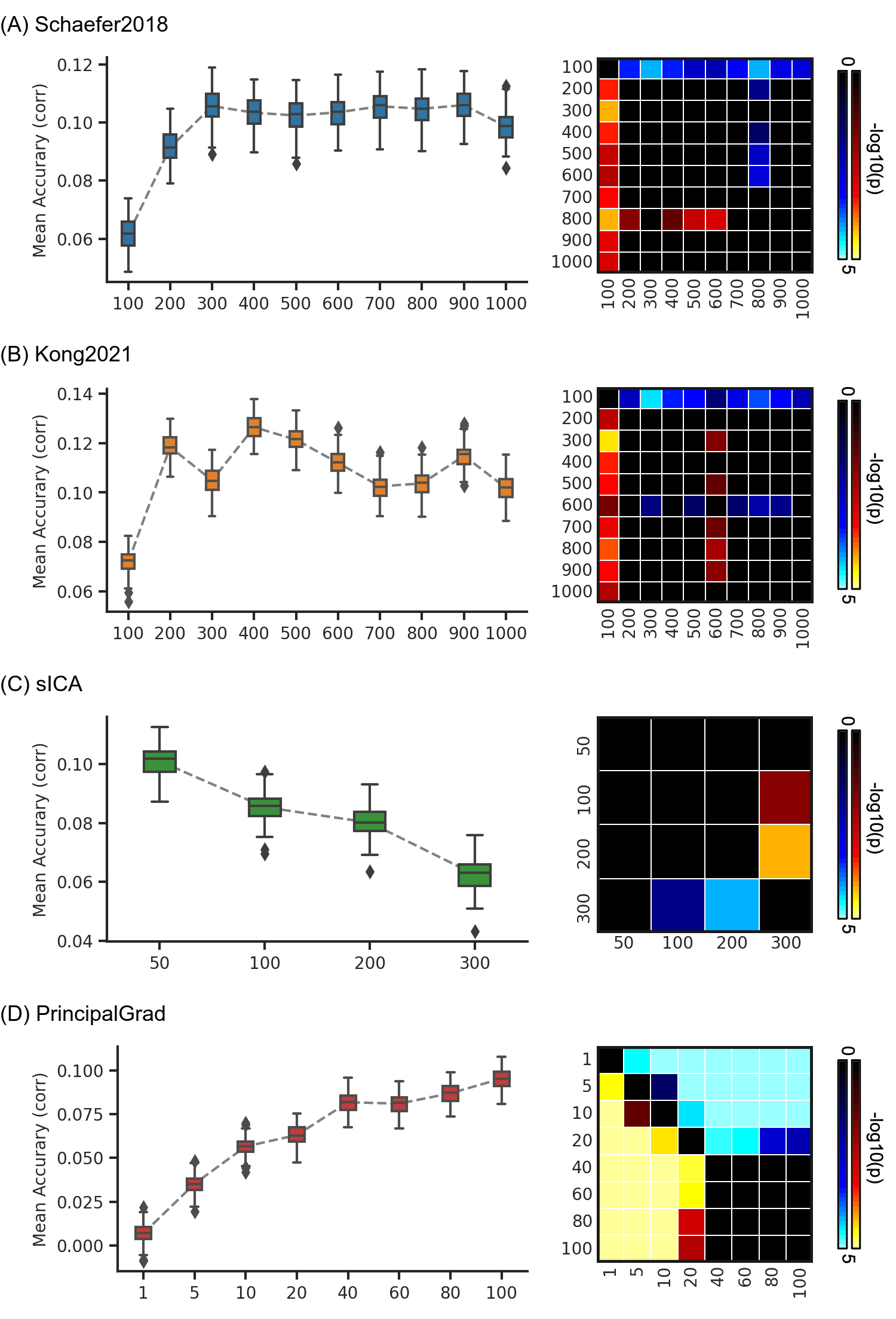


Figure S20. Average prediction accuracies (Pearson’s correlation) of self-reported measures vary across resolutions for gradient and parcellation approaches using KRR in the HCP dataset. (A) Prediction accuracies and p values of the hard-parcellation Schaefer2018 with 100 to 1000 ROIs. (B) Prediction accuracies and p values of the hard-parcellation Kong2021 with 100 to 1000 ROIs. (C) Prediction accuracies and p values of the soft-parcellation sICA with 50 to 300 components. (D) Prediction accuracies and p values of the principal gradient PrincipalGrad with 1 to 100 gradients. Boxplots utilized default Python seaborn parameters, that is, box shows median and interquartile range (IQR). Whiskers indicate 1.5 IQR. P values (-log10(p)) were computed between prediction accuracies of each pair of resolutions. Non-black colors denote significantly different prediction performances after correcting for multiple comparisons with FDR q < 0.05. Bright colors indicate small p values, dark colors indicate large p values. For each pair of comparisons, warm colors represent higher prediction accuracies of the “row” resolution than the “column” resolution.


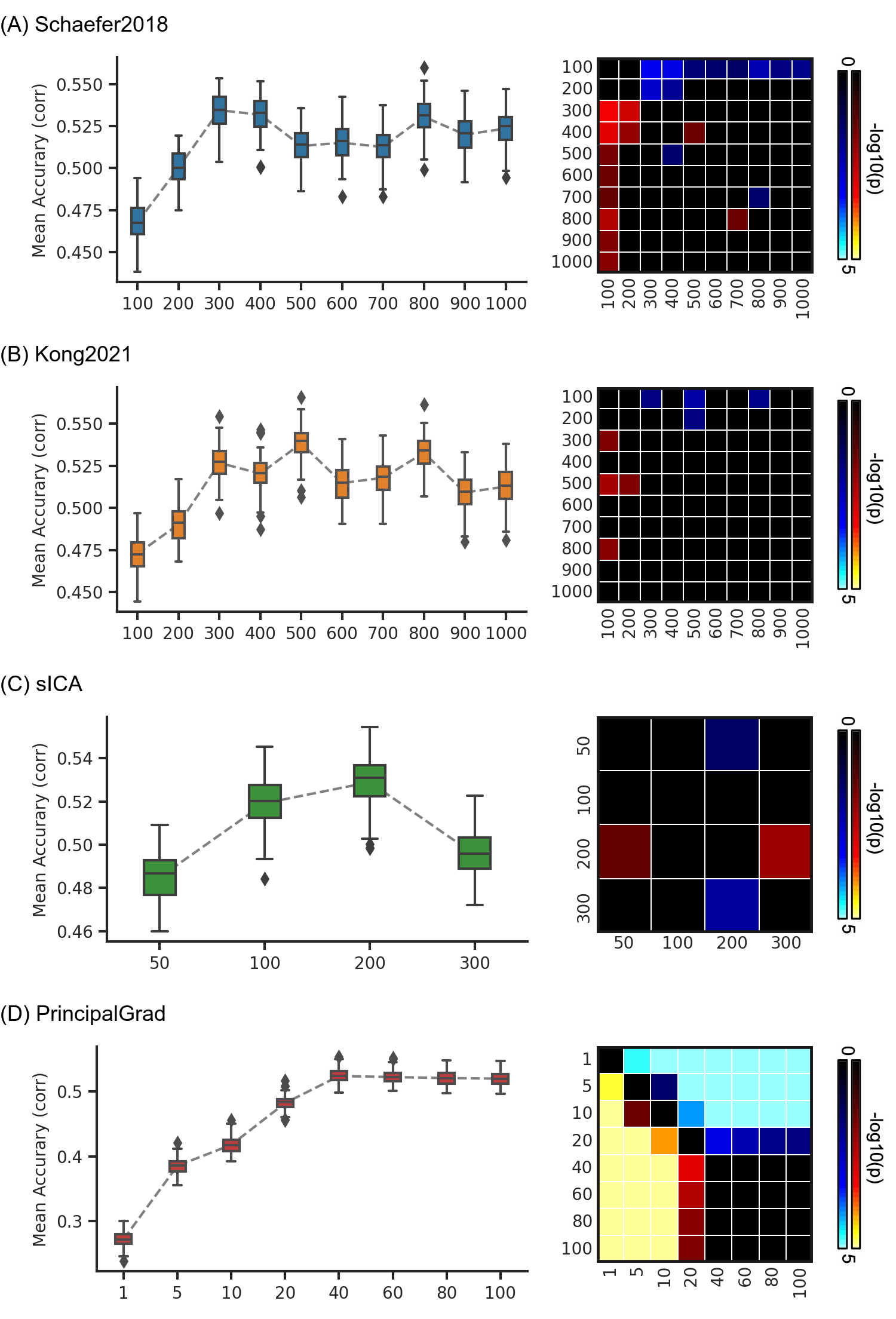


Figure S21. Prediction accuracies (Pearson’s correlation) of cognition vary across resolutions for gradient and parcellation approaches using KRR in the HCP dataset. (A) Prediction accuracies and p values of the hard-parcellation Schaefer2018 with 100 to 1000 ROIs. (B) Prediction accuracies and p values of the hard-parcellation Kong2021 with 100 to 1000 ROIs. (C) Prediction accuracies and p values of the soft-parcellation sICA with 50 to 300 components. (D) Prediction accuracies and p values of the principal gradient PrincipalGrad with 1 to 100 gradients. Boxplots utilized default Python seaborn parameters, that is, box shows median and interquartile range (IQR). Whiskers indicate 1.5 IQR. P values (-log10(p)) were computed between prediction accuracies of each pair of resolutions. Non-black colors denote significantly different prediction performances after correcting for multiple comparisons with FDR q < 0.05. Bright colors indicate small p values, dark colors indicate large p values. For each pair of comparisons, warm colors represent higher prediction accuracies of the “row” resolution than the “column” resolution.


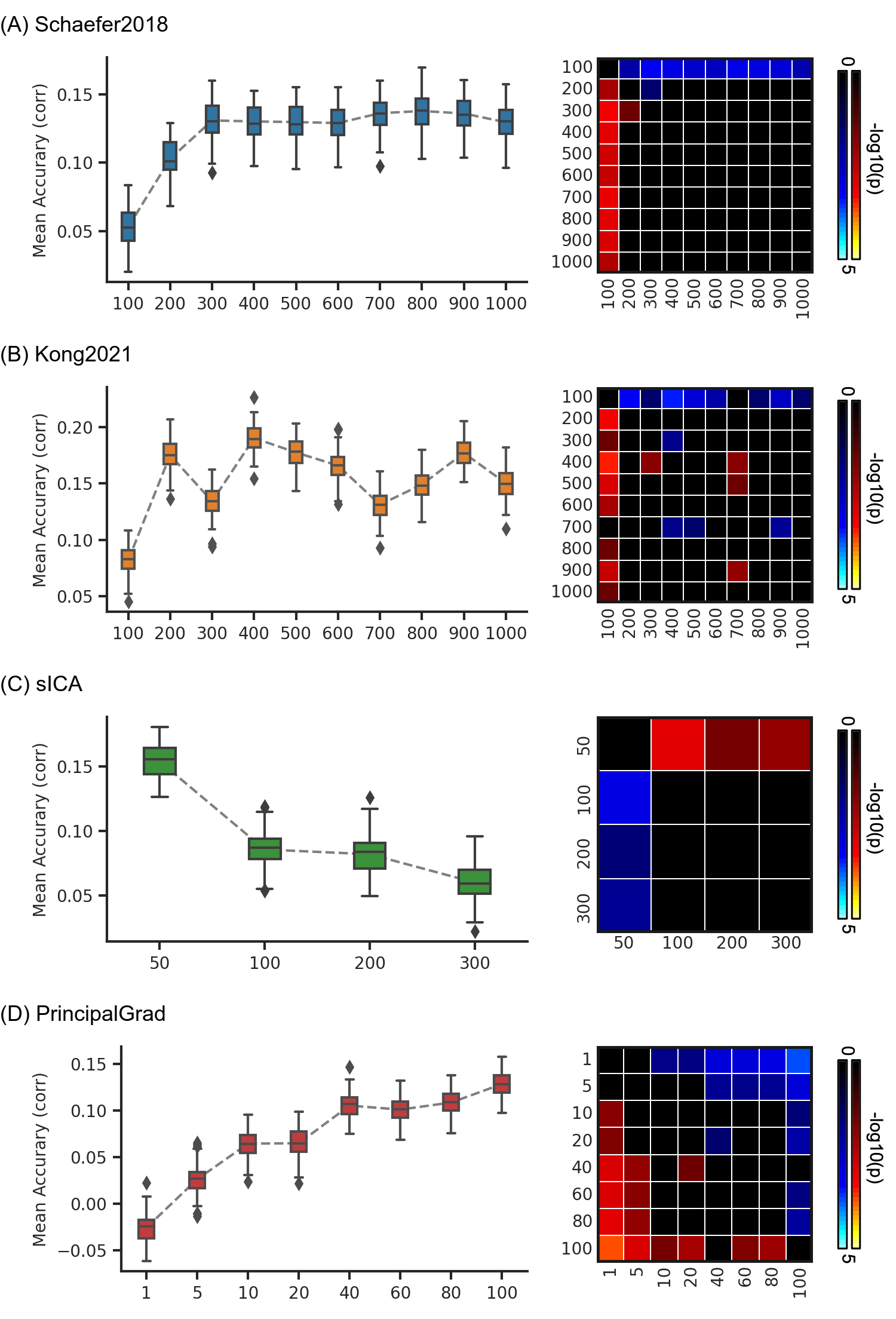


Figure S22. Prediction accuracies (Pearson’s correlation) of dissatisfaction vary across resolutions for gradient and parcellation approaches using KRR in the HCP dataset. (A) Prediction accuracies and p values of the hard-parcellation Schaefer2018 with 100 to 1000 ROIs. (B) Prediction accuracies and p values of the hard-parcellation Kong2021 with 100 to 1000 ROIs. (C) Prediction accuracies and p values of the soft-parcellation sICA with 50 to 300 components. (D) Prediction accuracies and p values of the principal gradient PrincipalGrad with 1 to 100 gradients. Boxplots utilized default Python seaborn parameters, that is, box shows median and interquartile range (IQR). Whiskers indicate 1.5 IQR. P values (-log10(p)) were computed between prediction accuracies of each pair of resolutions. Non-black colors denote significantly different prediction performances after correcting for multiple comparisons with FDR q < 0.05. Bright colors indicate small p values, dark colors indicate large p values. For each pair of comparisons, warm colors represent higher prediction accuracies of the “row” resolution than the “column” resolution.


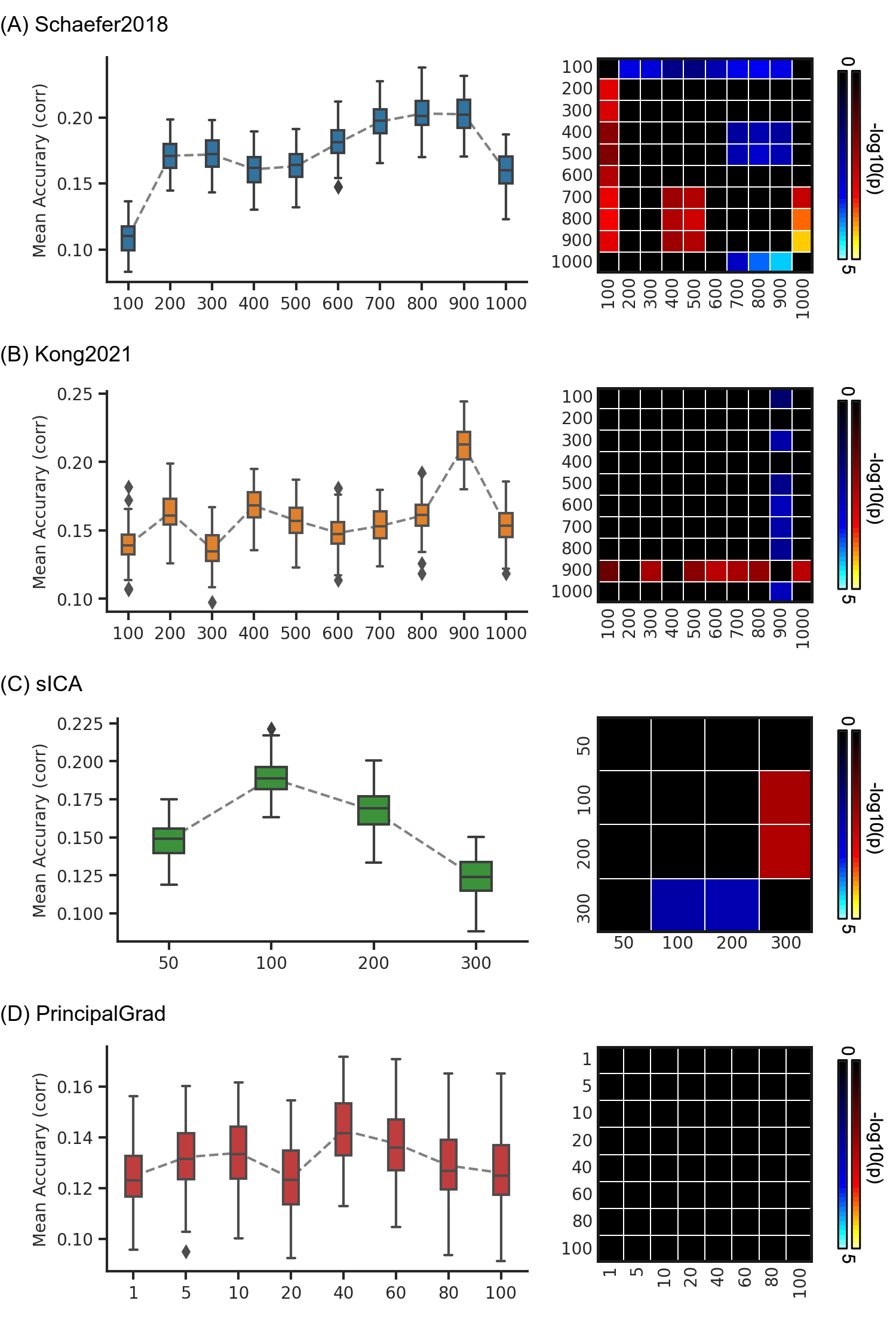


Figure S23. Prediction accuracies (Pearson’s correlation) of emotion vary across resolutions for gradient and parcellation approaches using KRR in the HCP dataset. (A) Prediction accuracies and p values of the hard-parcellation Schaefer2018 with 100 to 1000 ROIs. (B) Prediction accuracies and p values of the hard-parcellation Kong2021 with 100 to 1000 ROIs. (C) Prediction accuracies and p values of the soft-parcellation sICA with 50 to 300 components. (D) Prediction accuracies and p values of the principal gradient PrincipalGrad with 1 to 100 gradients. Boxplots utilized default Python seaborn parameters, that is, box shows median and interquartile range (IQR). Whiskers indicate 1.5 IQR. P values (-log10(p)) were computed between prediction accuracies of each pair of resolutions. Non-black colors denote significantly different prediction performances after correcting for multiple comparisons with FDR q < 0.05. Bright colors indicate small p values, dark colors indicate large p values. For each pair of comparisons, warm colors represent higher prediction accuracies of the “row” resolution than the “column” resolution.


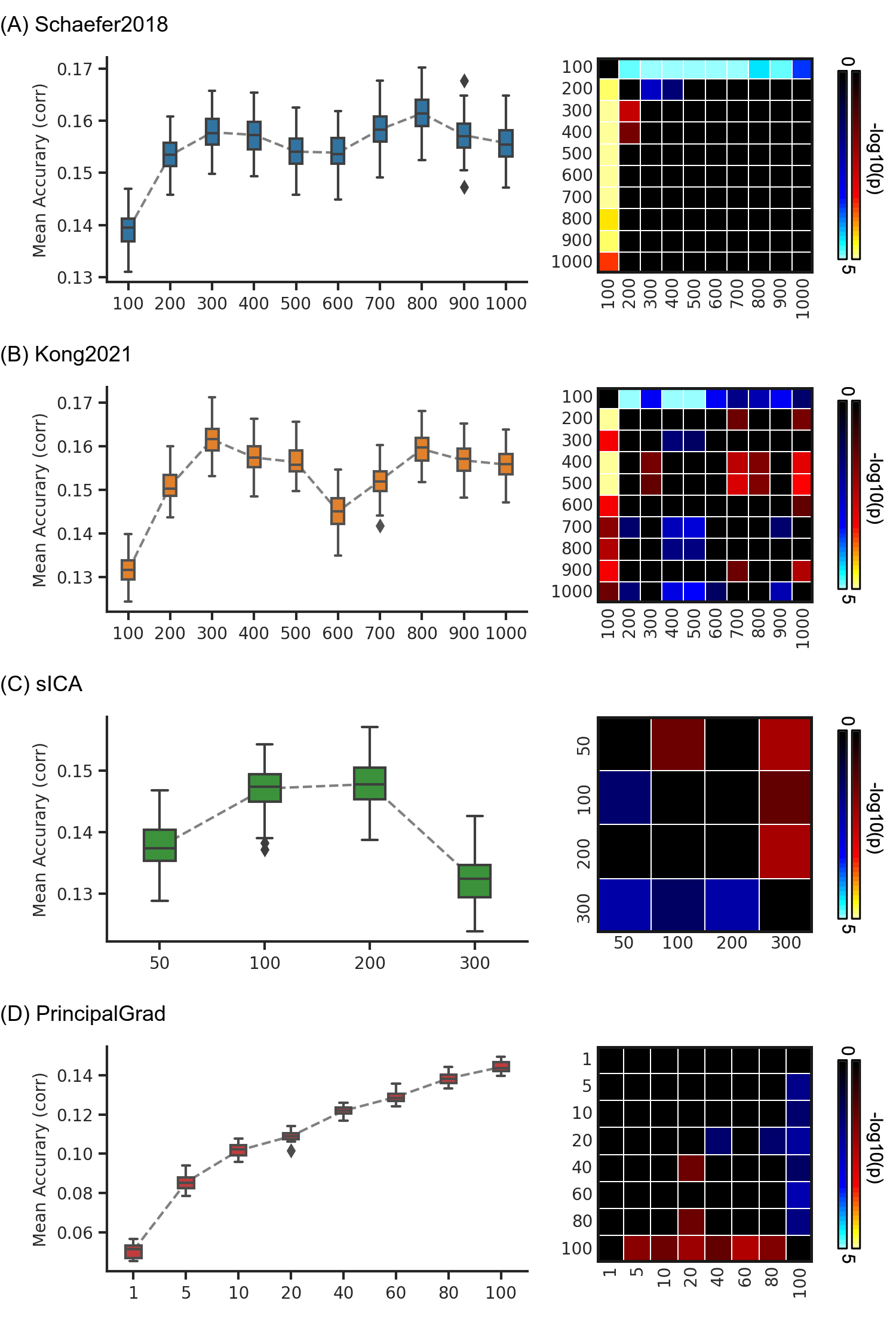


Figure S24. Average prediction accuracies (Pearson’s correlation) of task performance measures vary across resolutions for gradient and parcellation approaches using LRR in the HCP dataset. (A) Prediction accuracies and p values of the hard-parcellation Schaefer2018 with 100 to 1000 ROIs. (B) Prediction accuracies and p values of the hard-parcellation Kong2021 with 100 to 1000 ROIs. (C) Prediction accuracies and p values of the soft-parcellation sICA with 50 to 300 components. (D) Prediction accuracies and p values of the principal gradient PrincipalGrad with 1 to 100 gradients. Boxplots utilized default Python seaborn parameters, that is, box shows median and interquartile range (IQR). Whiskers indicate 1.5 IQR. P values (-log10(p)) were computed between prediction accuracies of each pair of resolutions. Non-black colors denote significantly different prediction performances after correcting for multiple comparisons with FDR q < 0.05. Bright colors indicate small p values, dark colors indicate large p values. For each pair of comparisons, warm colors represent higher prediction accuracies of the “row” resolution than the “column” resolution.


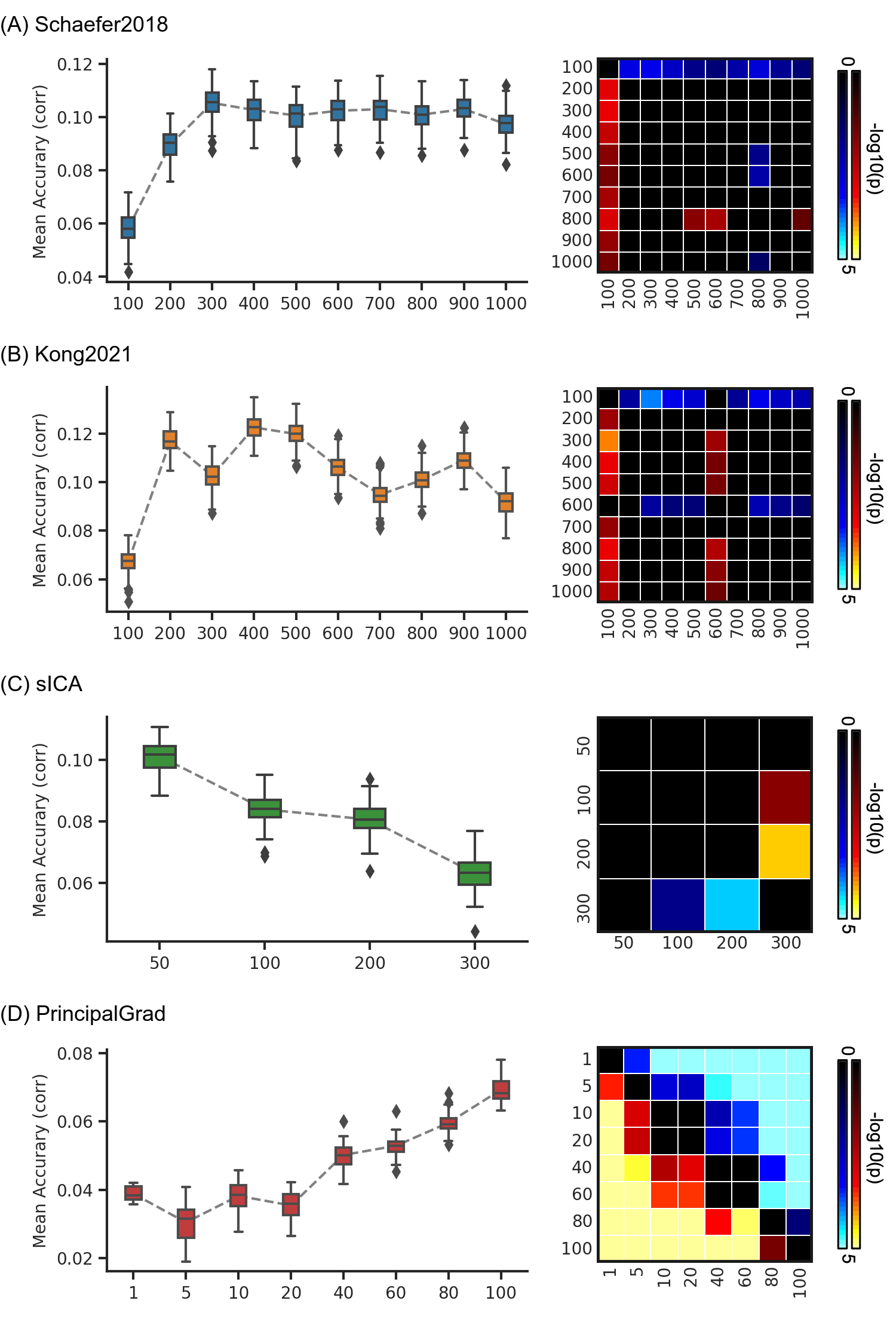


Figure S25. Average prediction accuracies (Pearson’s correlation) of self-reported measures vary across resolutions for gradient and parcellation approaches using LRR in the HCP dataset. (A) Prediction accuracies and p values of the hard-parcellation Schaefer2018 with 100 to 1000 ROIs. (B) Prediction accuracies and p values of the hard-parcellation Kong2021 with 100 to 1000 ROIs. (C) Prediction accuracies and p values of the soft-parcellation sICA with 50 to 300 components. (D) Prediction accuracies and p values of the principal gradient PrincipalGrad with 1 to 100 gradients. Boxplots utilized default Python seaborn parameters, that is, box shows median and interquartile range (IQR). Whiskers indicate 1.5 IQR. P values (-log10(p)) were computed between prediction accuracies of each pair of resolutions. Non-black colors denote significantly different prediction performances after correcting for multiple comparisons with FDR q < 0.05. Bright colors indicate small p values, dark colors indicate large p values. For each pair of comparisons, warm colors represent higher prediction accuracies of the “row” resolution than the “column” resolution.


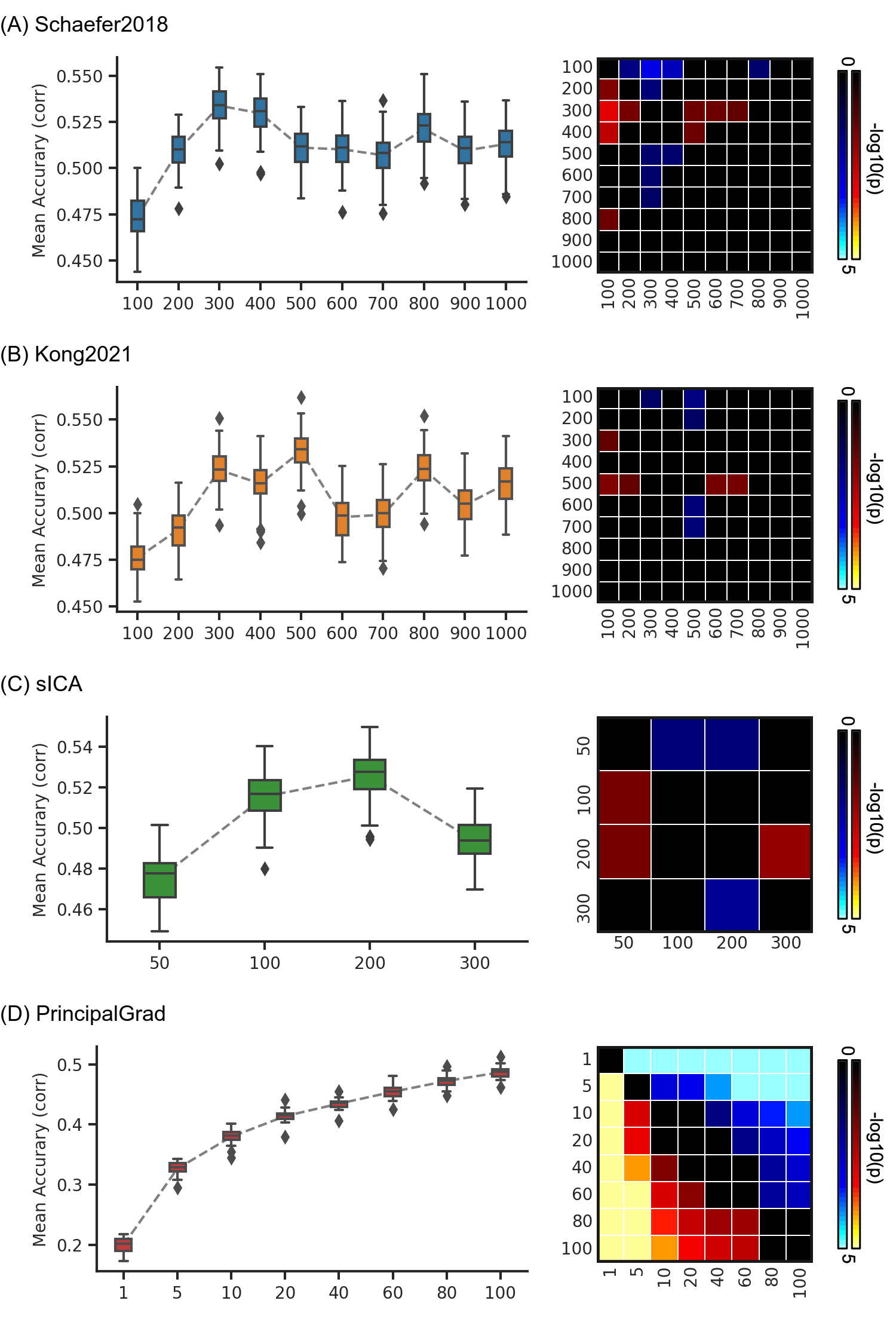


Figure S26. Prediction accuracies (Pearson’s correlation) of cognition vary across resolutions for gradient and parcellation approaches using LRR in the HCP dataset. (A) Prediction accuracies and p values of the hard-parcellation Schaefer2018 with 100 to 1000 ROIs. (B) Prediction accuracies and p values of the hard-parcellation Kong2021 with 100 to 1000 ROIs. (C) Prediction accuracies and p values of the soft-parcellation sICA with 50 to 300 components. (D) Prediction accuracies and p values of the principal gradient PrincipalGrad with 1 to 100 gradients. Boxplots utilized default Python seaborn parameters, that is, box shows median and interquartile range (IQR). Whiskers indicate 1.5 IQR. P values (-log10(p)) were computed between prediction accuracies of each pair of resolutions. Non-black colors denote significantly different prediction performances after correcting for multiple comparisons with FDR q < 0.05. Bright colors indicate small p values, dark colors indicate large p values. For each pair of comparisons, warm colors represent higher prediction accuracies of the “row” resolution than the “column” resolution.


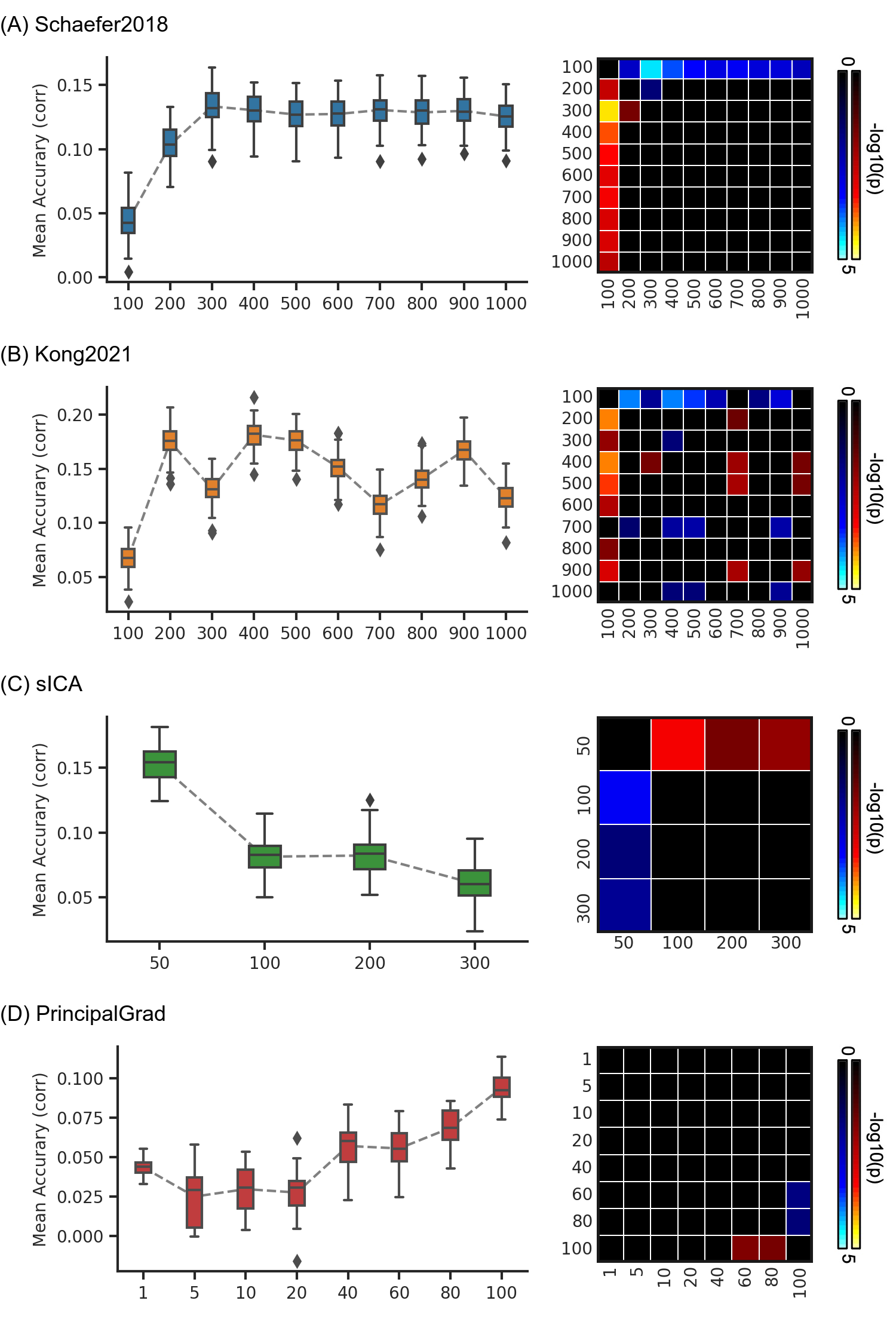


Figure S27. Prediction accuracies (Pearson’s correlation) of dissatisfaction vary across resolutions for gradient and parcellation approaches using LRR in the HCP dataset. (A) Prediction accuracies and p values of the hard-parcellation Schaefer2018 with 100 to 1000 ROIs. (B) Prediction accuracies and p values of the hard-parcellation Kong2021 with 100 to 1000 ROIs. (C) Prediction accuracies and p values of the soft-parcellation sICA with 50 to 300 components. (D) Prediction accuracies and p values of the principal gradient PrincipalGrad with 1 to 100 gradients. Boxplots utilized default Python seaborn parameters, that is, box shows median and interquartile range (IQR). Whiskers indicate 1.5 IQR. P values (-log10(p)) were computed between prediction accuracies of each pair of resolutions. Non-black colors denote significantly different prediction performances after correcting for multiple comparisons with FDR q < 0.05. Bright colors indicate small p values, dark colors indicate large p values. For each pair of comparisons, warm colors represent higher prediction accuracies of the “row” resolution than the “column” resolution.


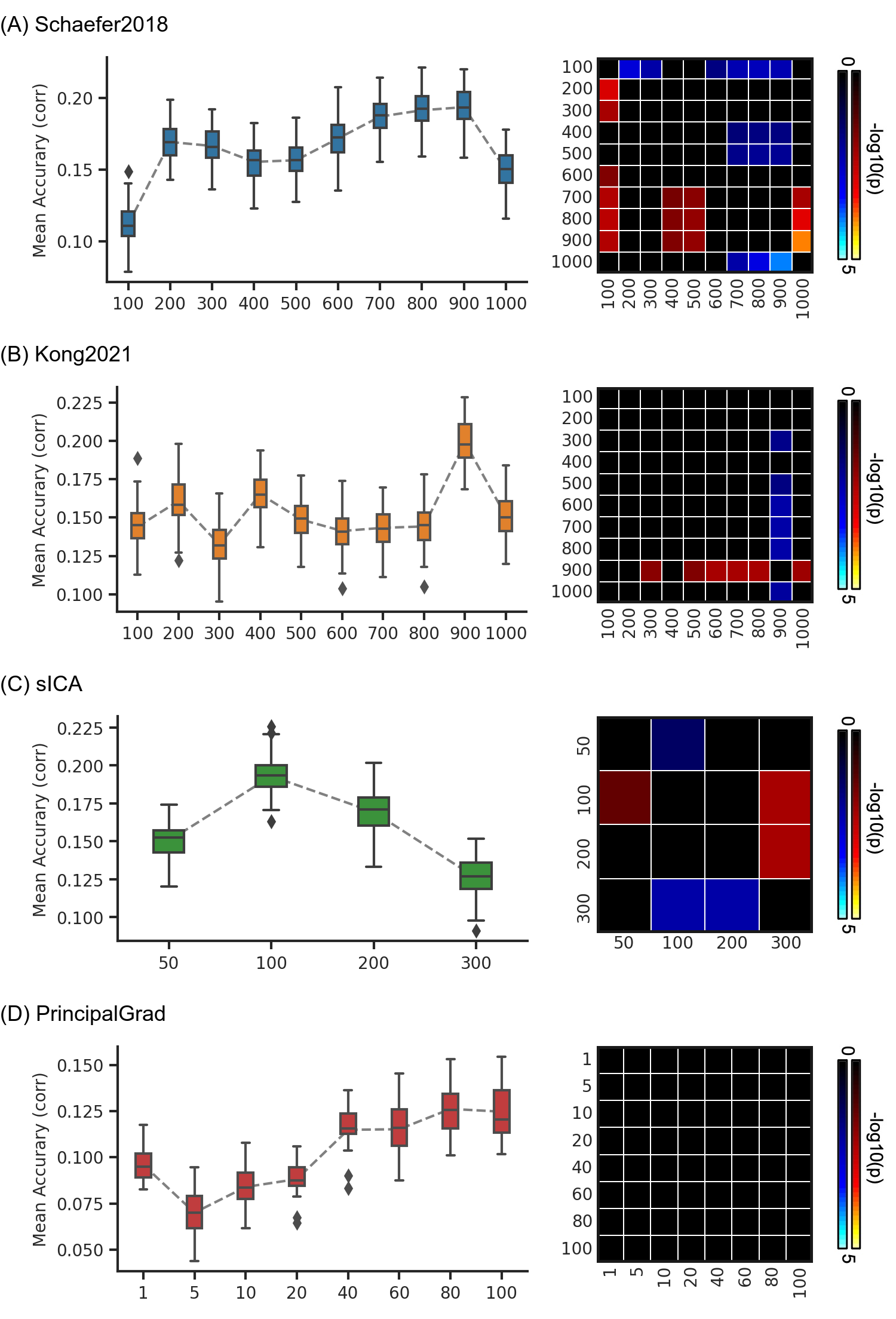


Figure S28. Prediction accuracies (Pearson’s correlation) of emotion vary across resolutions for gradient and parcellation approaches using LRR in the HCP dataset. (A) Prediction accuracies and p values of the hard-parcellation Schaefer2018 with 100 to 1000 ROIs. (B) Prediction accuracies and p values of the hard-parcellation Kong2021 with 100 to 1000 ROIs. (C) Prediction accuracies and p values of the soft-parcellation sICA with 50 to 300 components. (D) Prediction accuracies and p values of the principal gradient PrincipalGrad with 1 to 100 gradients. Boxplots utilized default Python seaborn parameters, that is, box shows median and interquartile range (IQR). Whiskers indicate 1.5 IQR. P values (-log10(p)) were computed between prediction accuracies of each pair of resolutions. Non-black colors denote significantly different prediction performances after correcting for multiple comparisons with FDR q < 0.05. Bright colors indicate small p values, dark colors indicate large p values. For each pair of comparisons, warm colors represent higher prediction accuracies of the “row” resolution than the “column” resolution.


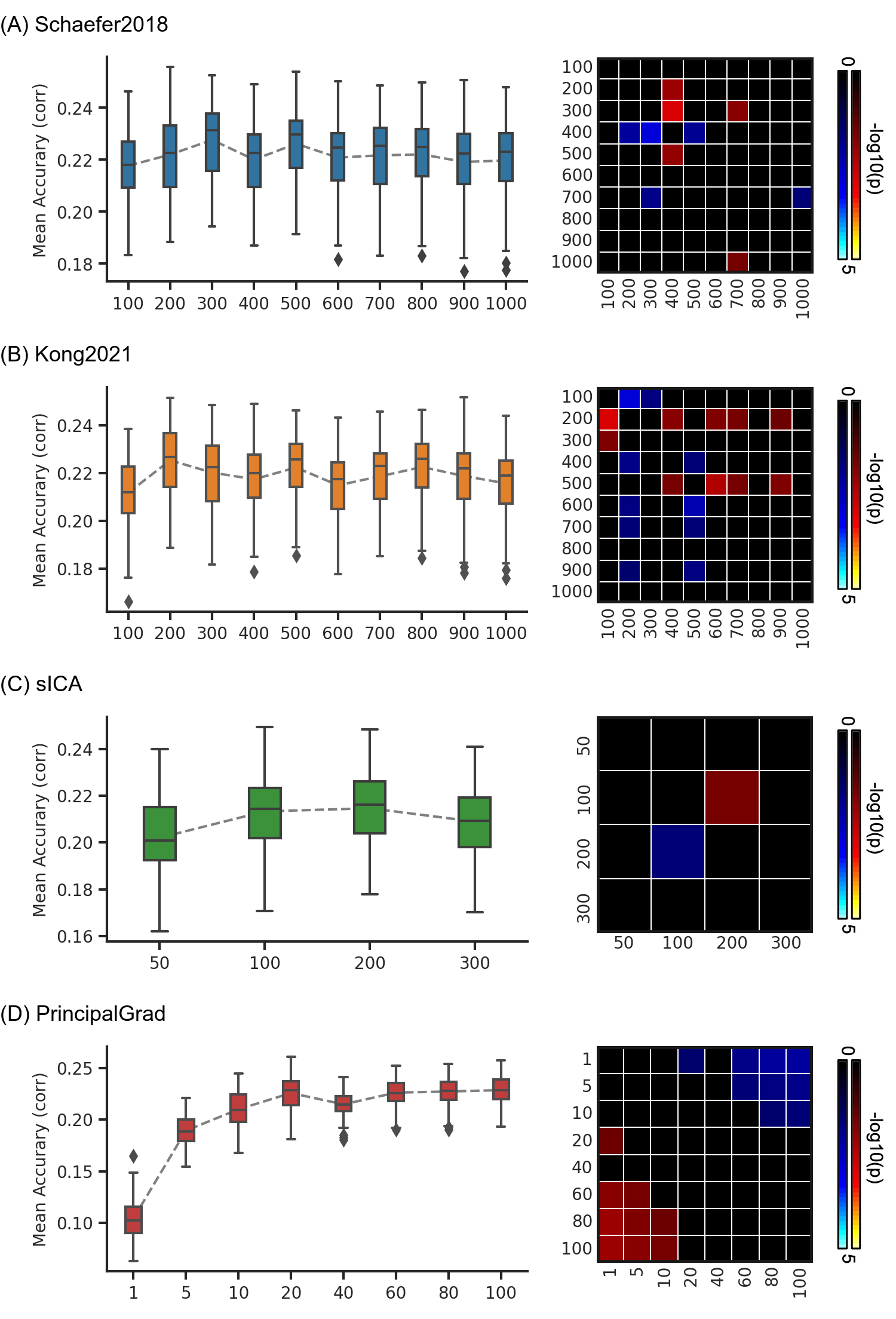


Figure S29. Average prediction accuracies (Pearson’s correlation) of task performance measures vary across resolutions for gradient and parcellation approaches using KRR in the ABCD dataset. (A) Prediction accuracies and p values of the hard-parcellation Schaefer2018 with 100 to 1000 ROIs. (B) Prediction accuracies and p values of the hard-parcellation Kong2021 with 100 to 1000 ROIs. (C) Prediction accuracies and p values of the soft-parcellation sICA with 50 to 300 components. (D) Prediction accuracies and p values of the principal gradient PrincipalGrad with 1 to 100 gradients. Boxplots utilized default Python seaborn parameters, that is, box shows median and interquartile range (IQR). Whiskers indicate 1.5 IQR. P values (-log10(p)) were computed between prediction accuracies of each pair of resolutions. Non-black colors denote significantly different prediction performances after correcting for multiple comparisons with FDR q < 0.05. Bright colors indicate small p values, dark colors indicate large p values. For each pair of comparisons, warm colors represent higher prediction accuracies of the “row” resolution than the “column” resolution.


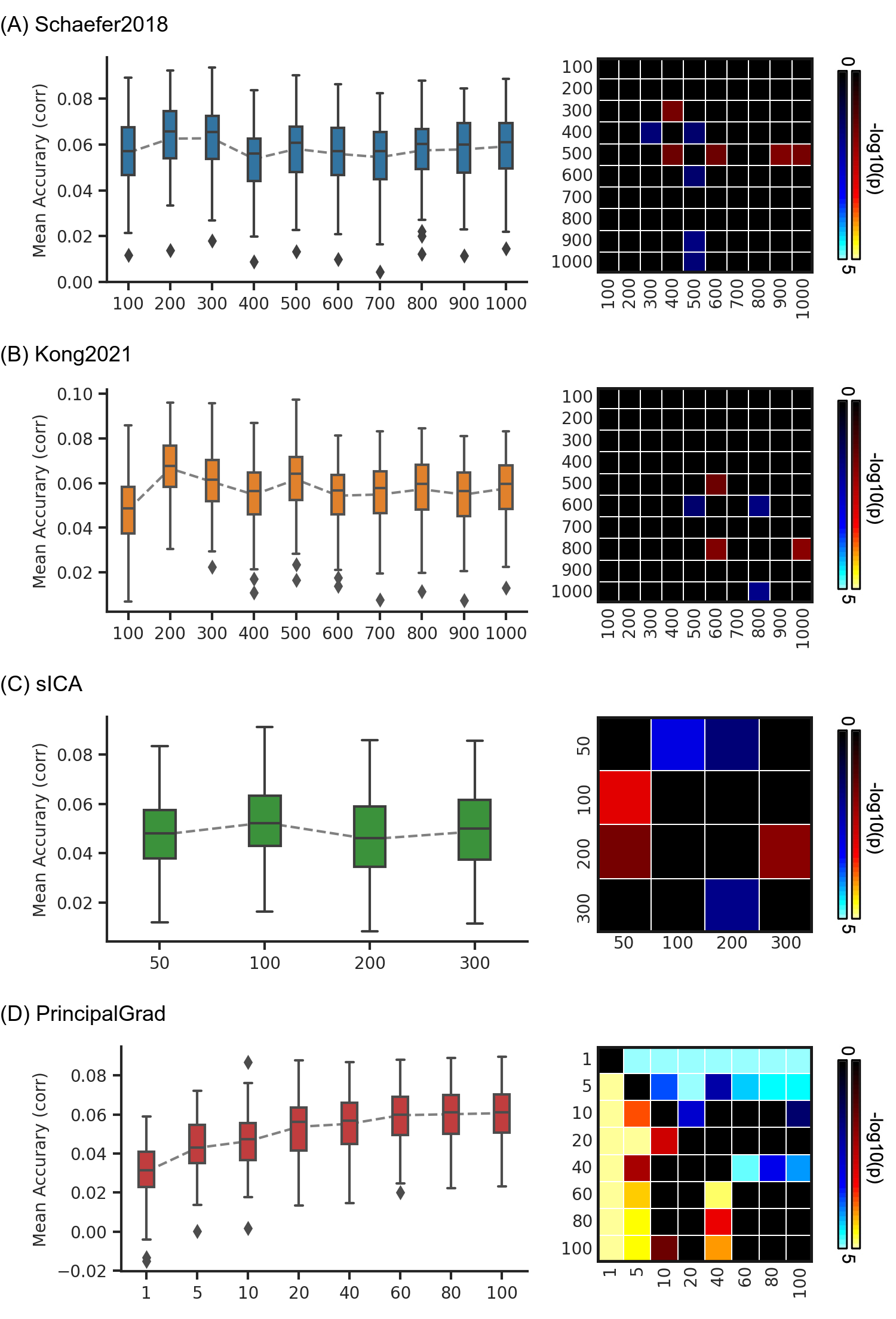


Figure S30. Average prediction accuracies (Pearson’s correlation) of self-reported measures vary across resolutions for gradient and parcellation approaches using KRR in the ABCD dataset. (A) Prediction accuracies and p values of the hard-parcellation Schaefer2018 with 100 to 1000 ROIs. (B) Prediction accuracies and p values of the hard-parcellation Kong2021 with 100 to 1000 ROIs. (C) Prediction accuracies and p values of the soft-parcellation sICA with 50 to 300 components. (D) Prediction accuracies and p values of the principal gradient PrincipalGrad with 1 to 100 gradients. Boxplots utilized default Python seaborn parameters, that is, box shows median and interquartile range (IQR). Whiskers indicate 1.5 IQR. P values (-log10(p)) were computed between prediction accuracies of each pair of resolutions. Non-black colors denote significantly different prediction performances after correcting for multiple comparisons with FDR q < 0.05. Bright colors indicate small p values, dark colors indicate large p values. For each pair of comparisons, warm colors represent higher prediction accuracies of the “row” resolution than the “column” resolution.


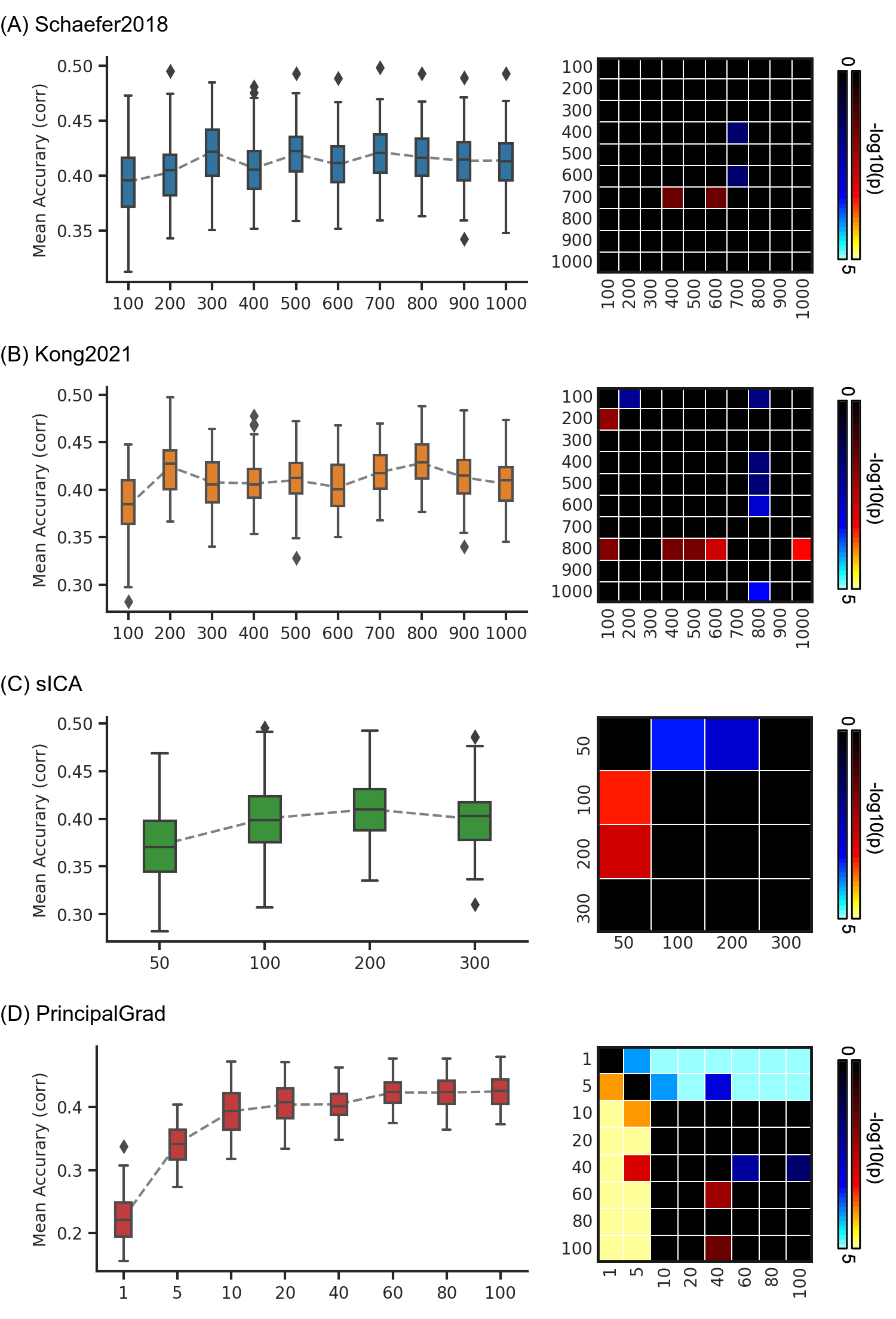


Figure S31. Prediction accuracies (Pearson’s correlation) of cognition vary across resolutions for gradient and parcellation approaches using KRR in the ABCD dataset. (A) Prediction accuracies and p values of the hard-parcellation Schaefer2018 with 100 to 1000 ROIs. (B) Prediction accuracies and p values of the hard-parcellation Kong2021 with 100 to 1000 ROIs. (C) Prediction accuracies and p values of the soft-parcellation sICA with 50 to 300 components. (D) Prediction accuracies and p values of the principal gradient PrincipalGrad with 1 to 100 gradients. Boxplots utilized default Python seaborn parameters, that is, box shows median and interquartile range (IQR). Whiskers indicate 1.5 IQR. P values (-log10(p)) were computed between prediction accuracies of each pair of resolutions. Non-black colors denote significantly different prediction performances after correcting for multiple comparisons with FDR q < 0.05. Bright colors indicate small p values, dark colors indicate large p values. For each pair of comparisons, warm colors represent higher prediction accuracies of the “row” resolution than the “column” resolution.


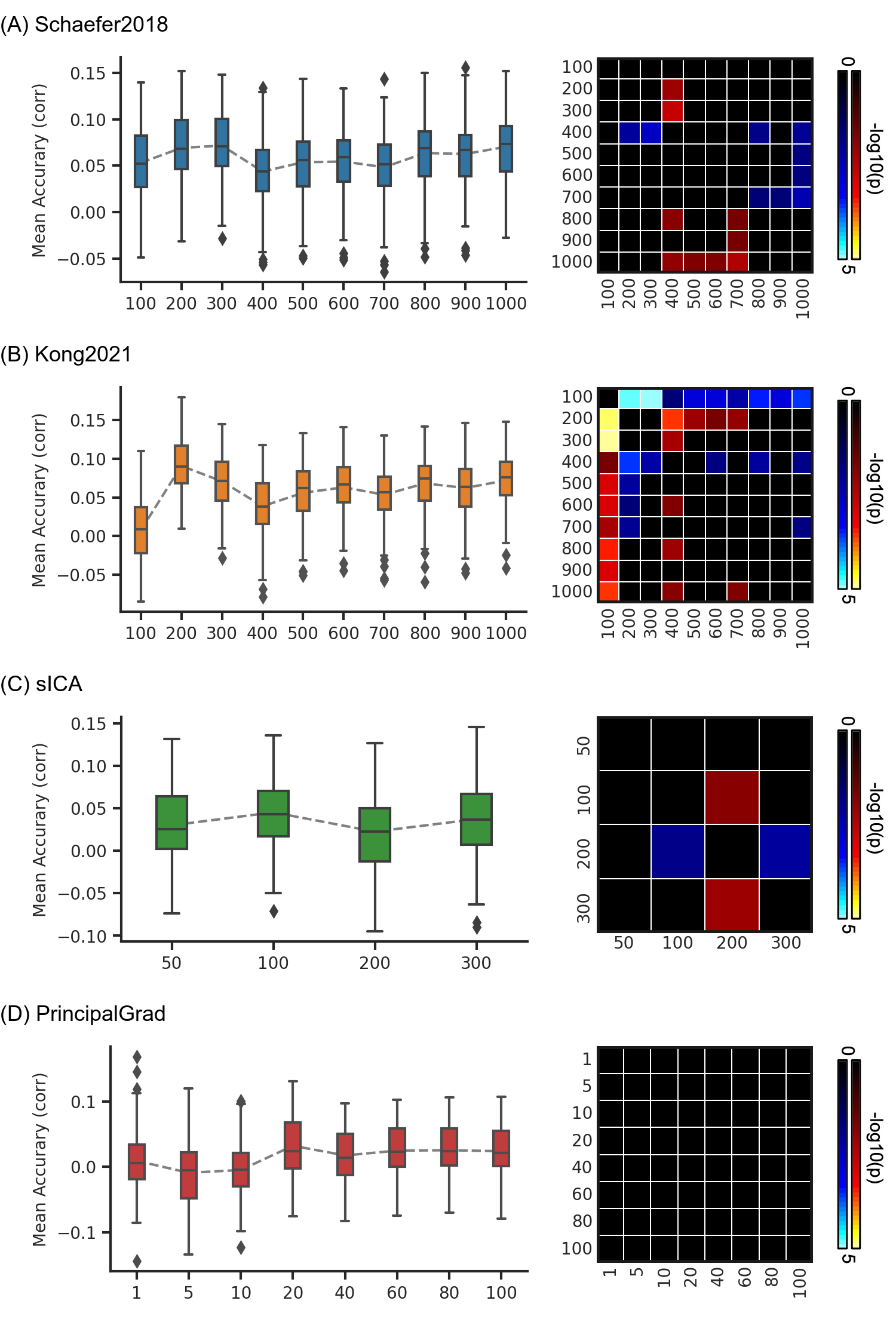


Figure S32. Prediction accuracies (Pearson’s correlation) of mental health vary across resolutions for gradient and parcellation approaches using KRR in the ABCD dataset. (A) Prediction accuracies and p values of the hard-parcellation Schaefer2018 with 100 to 1000 ROIs. (B) Prediction accuracies and p values of the hard-parcellation Kong2021 with 100 to 1000 ROIs. (C) Prediction accuracies and p values of the soft-parcellation sICA with 50 to 300 components. (D) Prediction accuracies and p values of the principal gradient PrincipalGrad with 1 to 100 gradients. Boxplots utilized default Python seaborn parameters, that is, box shows median and interquartile range (IQR). Whiskers indicate 1.5 IQR. P values (-log10(p)) were computed between prediction accuracies of each pair of resolutions. Non-black colors denote significantly different prediction performances after correcting for multiple comparisons with FDR q < 0.05. Bright colors indicate small p values, dark colors indicate large p values. For each pair of comparisons, warm colors represent higher prediction accuracies of the “row” resolution than the “column” resolution.


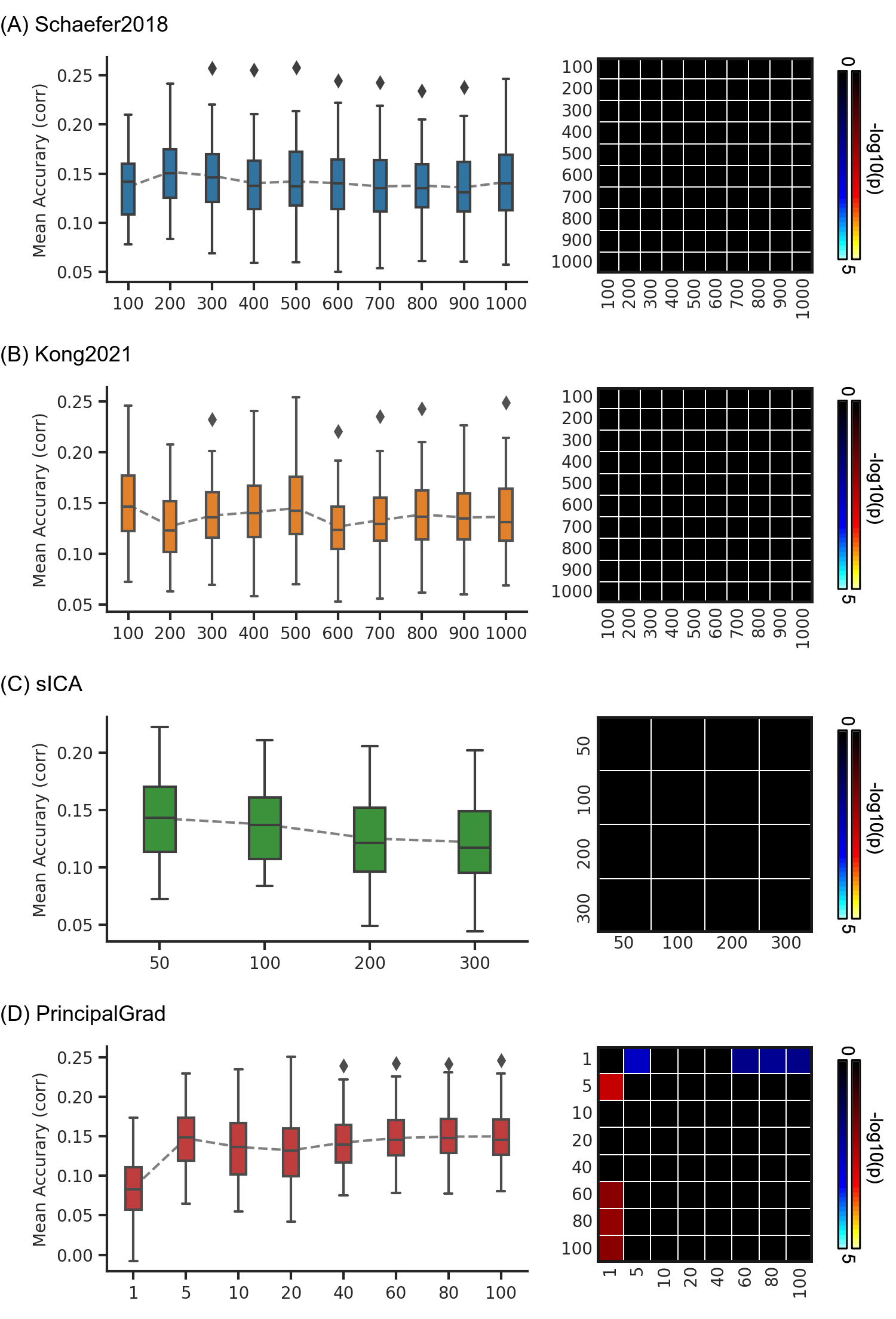


Figure S33. Prediction accuracies (Pearson’s correlation) of personality vary across resolutions for gradient and parcellation approaches using KRR in the ABCD dataset. (A) Prediction accuracies and p values of the hard-parcellation Schaefer2018 with 100 to 1000 ROIs. (B) Prediction accuracies and p values of the hard-parcellation Kong2021 with 100 to 1000 ROIs. (C) Prediction accuracies and p values of the soft-parcellation sICA with 50 to 300 components. (D) Prediction accuracies and p values of the principal gradient PrincipalGrad with 1 to 100 gradients. Boxplots utilized default Python seaborn parameters, that is, box shows median and interquartile range (IQR). Whiskers indicate 1.5 IQR. P values (-log10(p)) were computed between prediction accuracies of each pair of resolutions. Non-black colors denote significantly different prediction performances after correcting for multiple comparisons with FDR q < 0.05. Bright colors indicate small p values, dark colors indicate large p values. For each pair of comparisons, warm colors represent higher prediction accuracies of the “row” resolution than the “column” resolution.


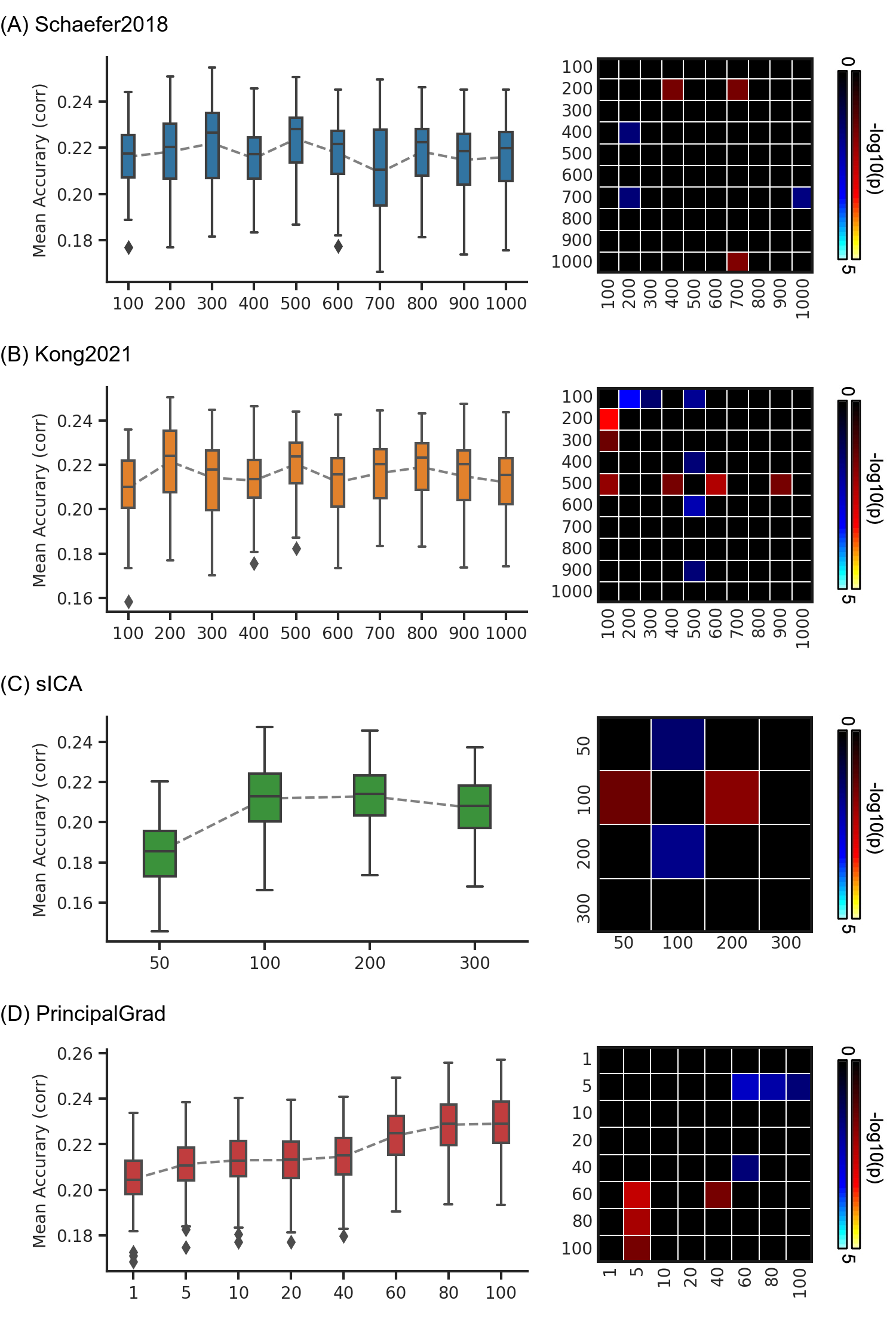


Figure S34. Average prediction accuracies (Pearson’s correlation) of task performance measures vary across resolutions for gradient and parcellation approaches using LRR in the ABCD dataset. (A) Prediction accuracies and p values of the hard-parcellation Schaefer2018 with 100 to 1000 ROIs. (B) Prediction accuracies and p values of the hard-parcellation Kong2021 with 100 to 1000 ROIs. (C) Prediction accuracies and p values of the soft-parcellation sICA with 50 to 300 components. (D) Prediction accuracies and p values of the principal gradient PrincipalGrad with 1 to 100 gradients. Boxplots utilized default Python seaborn parameters, that is, box shows median and interquartile range (IQR). Whiskers indicate 1.5 IQR. P values (-log10(p)) were computed between prediction accuracies of each pair of resolutions. Non-black colors denote significantly different prediction performances after correcting for multiple comparisons with FDR q < 0.05. Bright colors indicate small p values, dark colors indicate large p values. For each pair of comparisons, warm colors represent higher prediction accuracies of the “row” resolution than the “column” resolution.


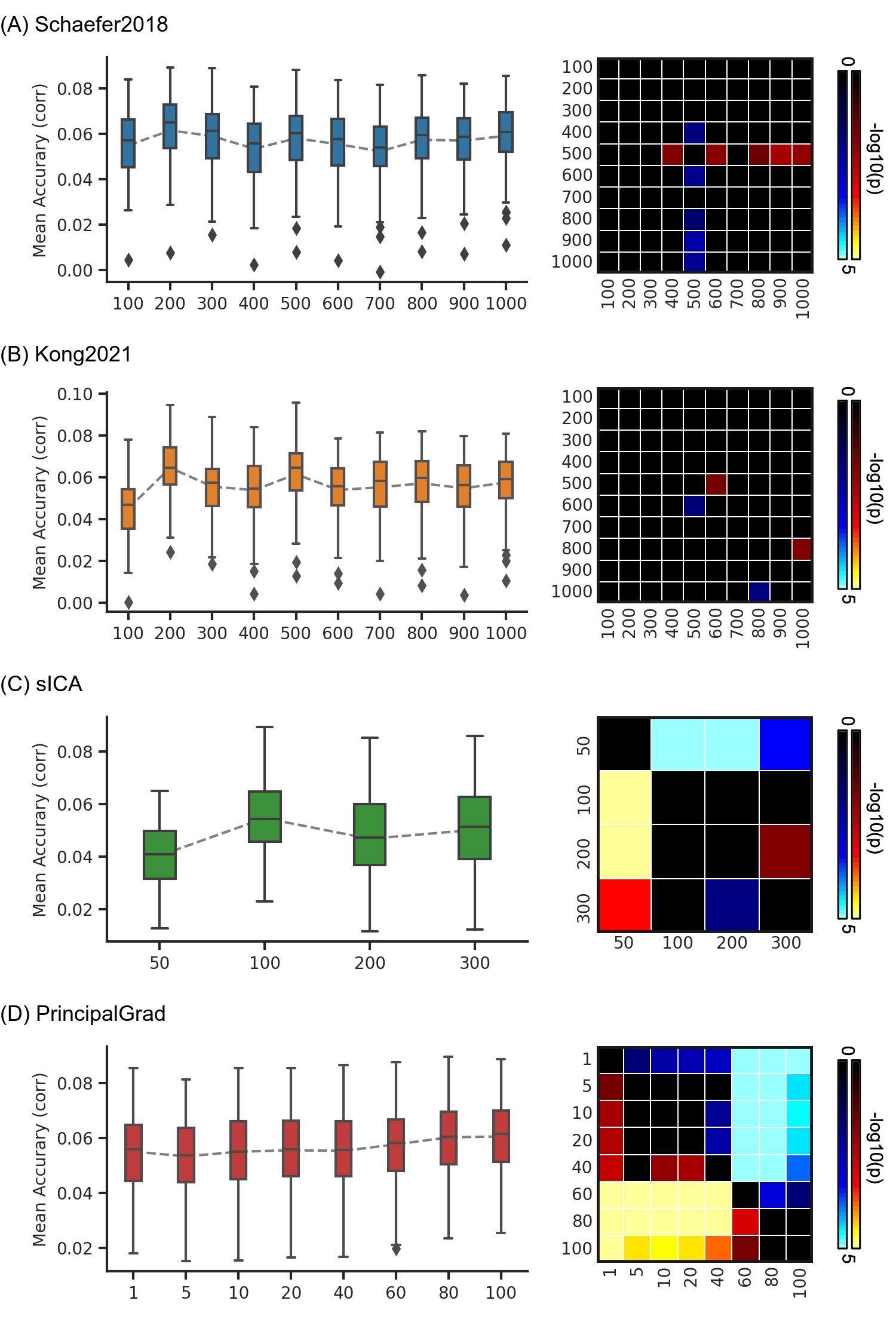


Figure S35. Average prediction accuracies (Pearson’s correlation) of self-reported measures vary across resolutions for gradient and parcellation approaches using LRR in the ABCD dataset. (A) Prediction accuracies and p values of the hard-parcellation Schaefer2018 with 100 to 1000 ROIs. (B) Prediction accuracies and p values of the hard-parcellation Kong2021 with 100 to 1000 ROIs. (C) Prediction accuracies and p values of the soft-parcellation sICA with 50 to 300 components. (D) Prediction accuracies and p values of the principal gradient PrincipalGrad with 1 to 100 gradients. Boxplots utilized default Python seaborn parameters, that is, box shows median and interquartile range (IQR). Whiskers indicate 1.5 IQR. P values (-log10(p)) were computed between prediction accuracies of each pair of resolutions. Non-black colors denote significantly different prediction performances after correcting for multiple comparisons with FDR q < 0.05. Bright colors indicate small p values, dark colors indicate large p values. For each pair of comparisons, warm colors represent higher prediction accuracies of the “row” resolution than the “column” resolution.


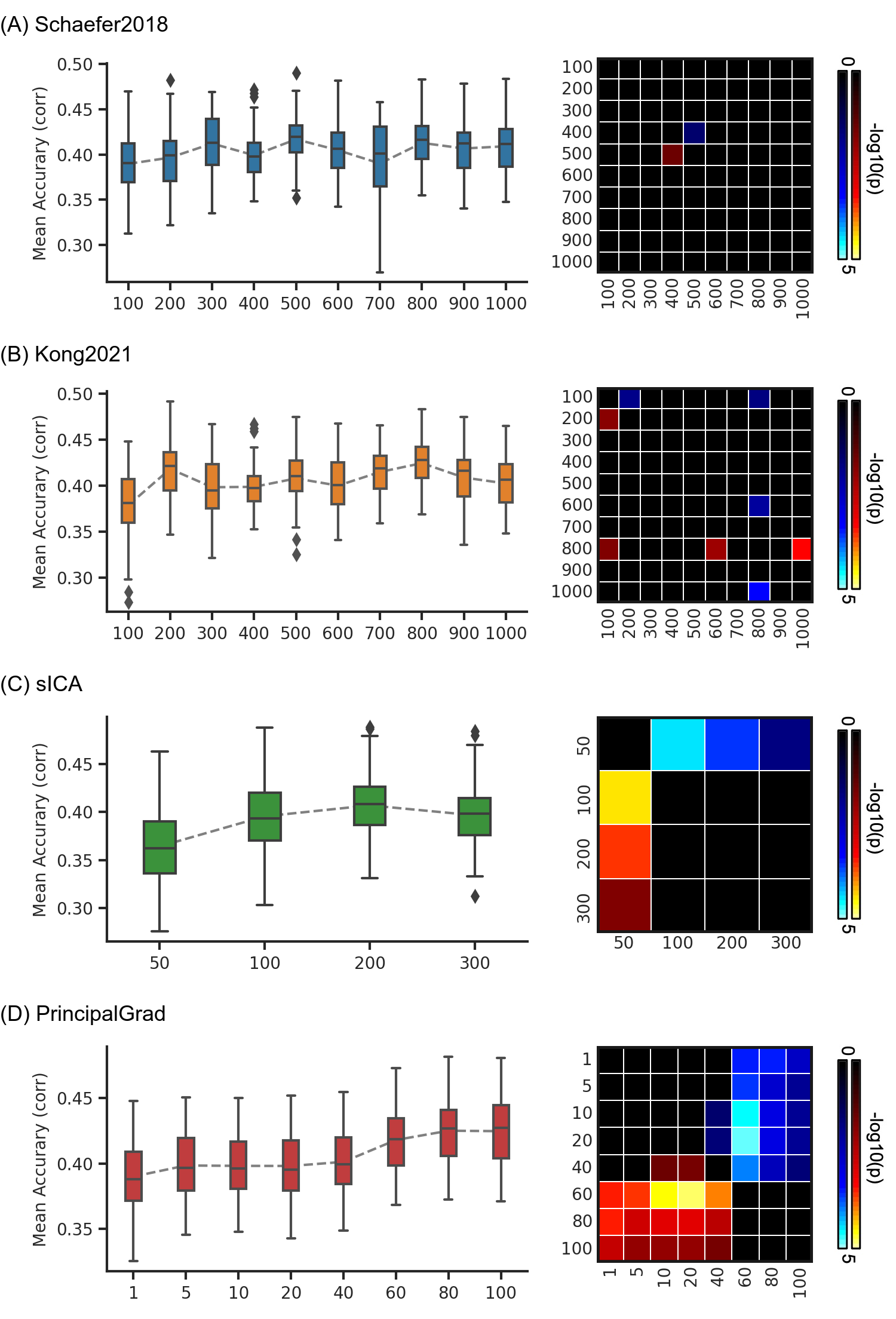


Figure S36. Prediction accuracies (Pearson’s correlation) of cognition vary across resolutions for gradient and parcellation approaches using LRR in the ABCD dataset. (A) Prediction accuracies and p values of the hard-parcellation Schaefer2018 with 100 to 1000 ROIs. (B) Prediction accuracies and p values of the hard-parcellation Kong2021 with 100 to 1000 ROIs. (C) Prediction accuracies and p values of the soft-parcellation sICA with 50 to 300 components. (D) Prediction accuracies and p values of the principal gradient PrincipalGrad with 1 to 100 gradients. Boxplots utilized default Python seaborn parameters, that is, box shows median and interquartile range (IQR). Whiskers indicate 1.5 IQR. P values (-log10(p)) were computed between prediction accuracies of each pair of resolutions. Non-black colors denote significantly different prediction performances after correcting for multiple comparisons with FDR q < 0.05. Bright colors indicate small p values, dark colors indicate large p values. For each pair of comparisons, warm colors represent higher prediction accuracies of the “row” resolution than the “column” resolution.


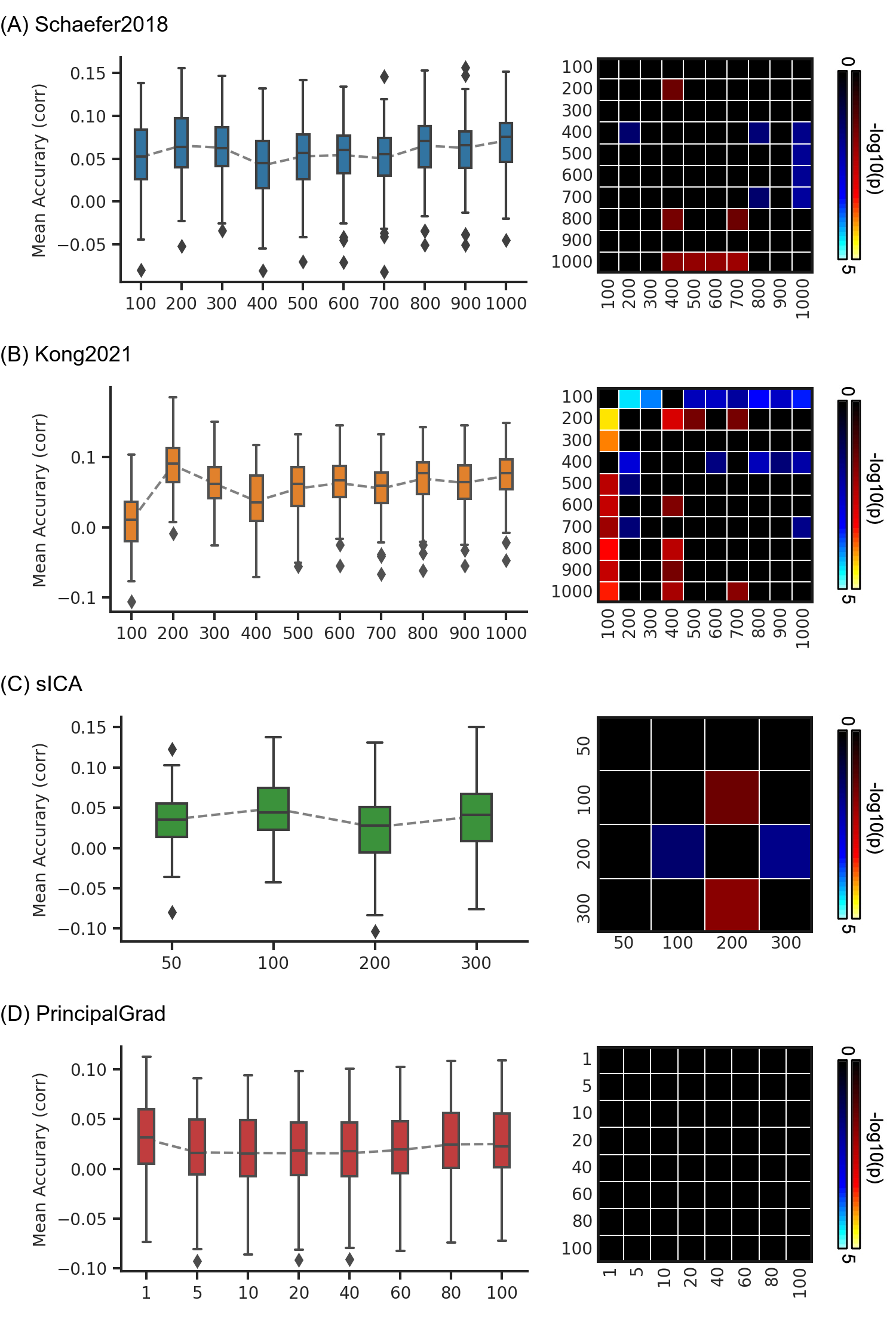


Figure S37. Prediction accuracies (Pearson’s correlation) of mental health vary across resolutions for gradient and parcellation approaches using LRR in the ABCD dataset. (A) Prediction accuracies and p values of the hard-parcellation Schaefer2018 with 100 to 1000 ROIs. (B) Prediction accuracies and p values of the hard-parcellation Kong2021 with 100 to 1000 ROIs. (C) Prediction accuracies and p values of the soft-parcellation sICA with 50 to 300 components. (D) Prediction accuracies and p values of the principal gradient PrincipalGrad with 1 to 100 gradients. Boxplots utilized default Python seaborn parameters, that is, box shows median and interquartile range (IQR). Whiskers indicate 1.5 IQR. P values (-log10(p)) were computed between prediction accuracies of each pair of resolutions. Non-black colors denote significantly different prediction performances after correcting for multiple comparisons with FDR q < 0.05. Bright colors indicate small p values, dark colors indicate large p values. For each pair of comparisons, warm colors represent higher prediction accuracies of the “row” resolution than the “column” resolution.


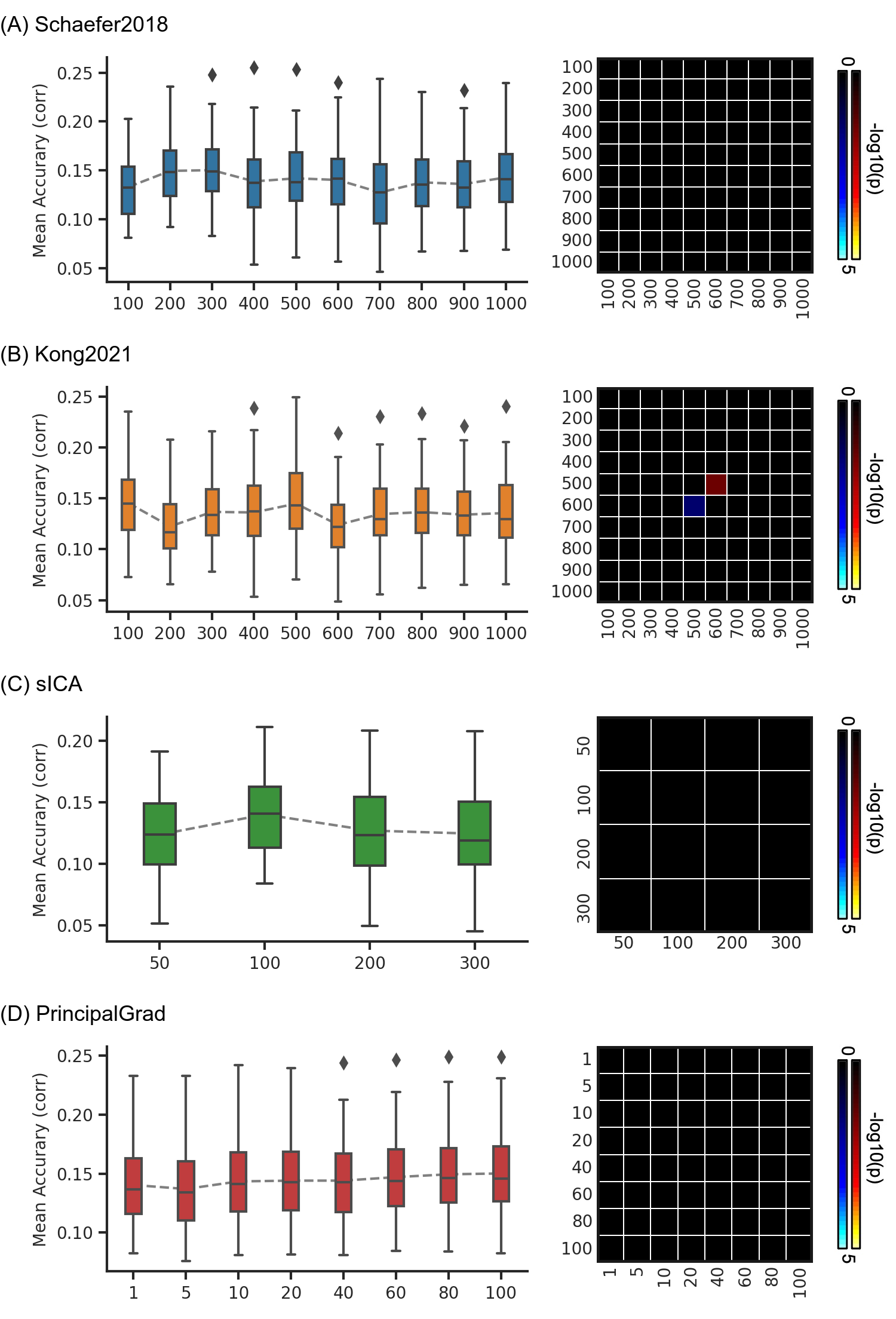


Figure S38. Prediction accuracies (Pearson’s correlation) of emotion vary across resolutions for gradient and parcellation approaches using LRR in the ABCD dataset. (A) Prediction accuracies and p values of the hard-parcellation Schaefer2018 with 100 to 1000 ROIs. (B) Prediction accuracies and p values of the hard-parcellation Kong2021 with 100 to 1000 ROIs. (C) Prediction accuracies and p values of the soft-parcellation sICA with 50 to 300 components. (D) Prediction accuracies and p values of the principal gradient PrincipalGrad with 1 to 100 gradients. Boxplots utilized default Python seaborn parameters, that is, box shows median and interquartile range (IQR). Whiskers indicate 1.5 IQR. P values (-log10(p)) were computed between prediction accuracies of each pair of resolutions. Non-black colors denote significantly different prediction performances after correcting for multiple comparisons with FDR q < 0.05. Bright colors indicate small p values, dark colors indicate large p values. For each pair of comparisons, warm colors represent higher prediction accuracies of the “row” resolution than the “column” resolution.


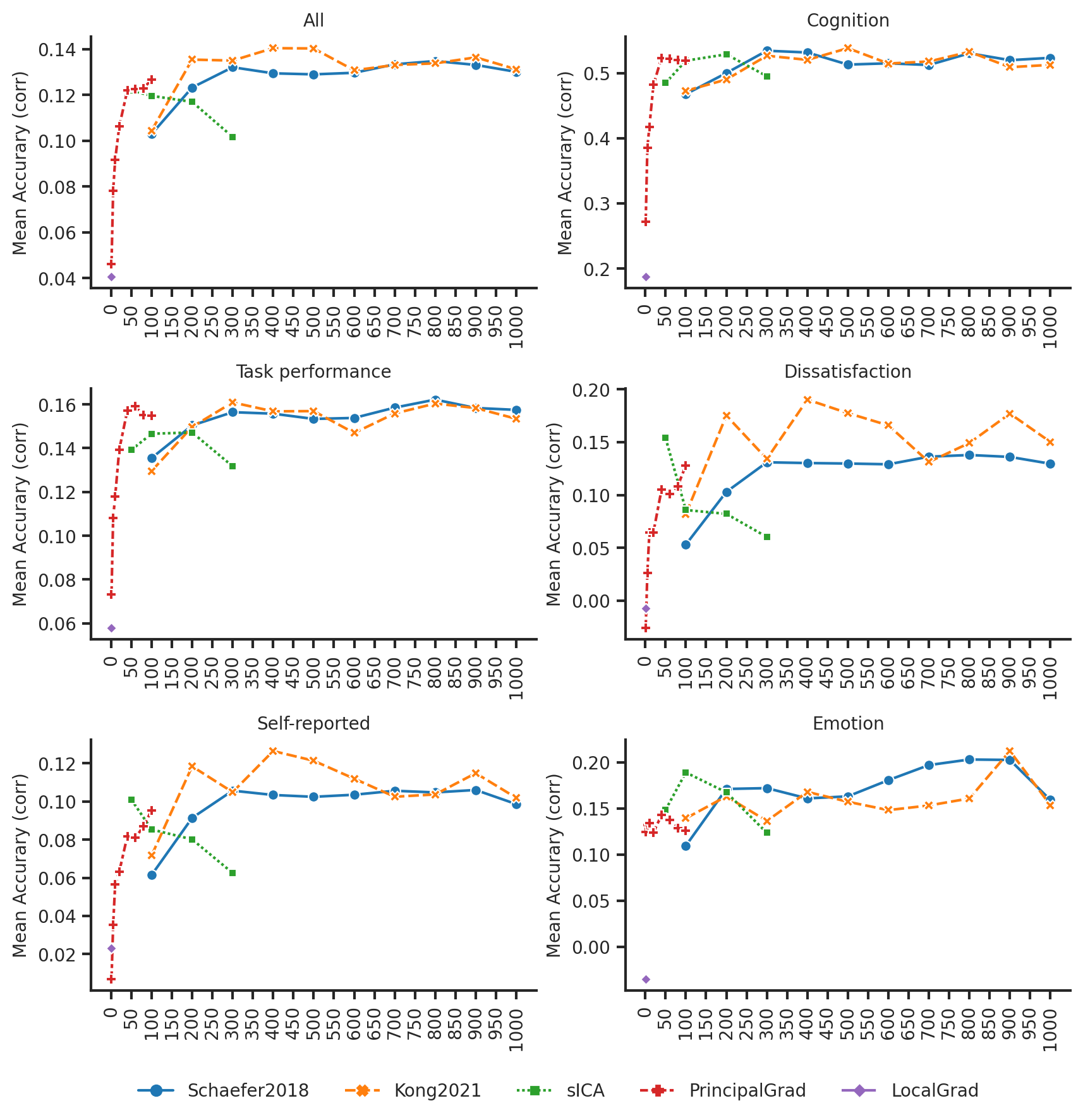


Figure S39. Prediction accuracies (Pearson’s correlation) vary across resolutions for gradient and parcellation approaches using KRR in the HCP dataset.


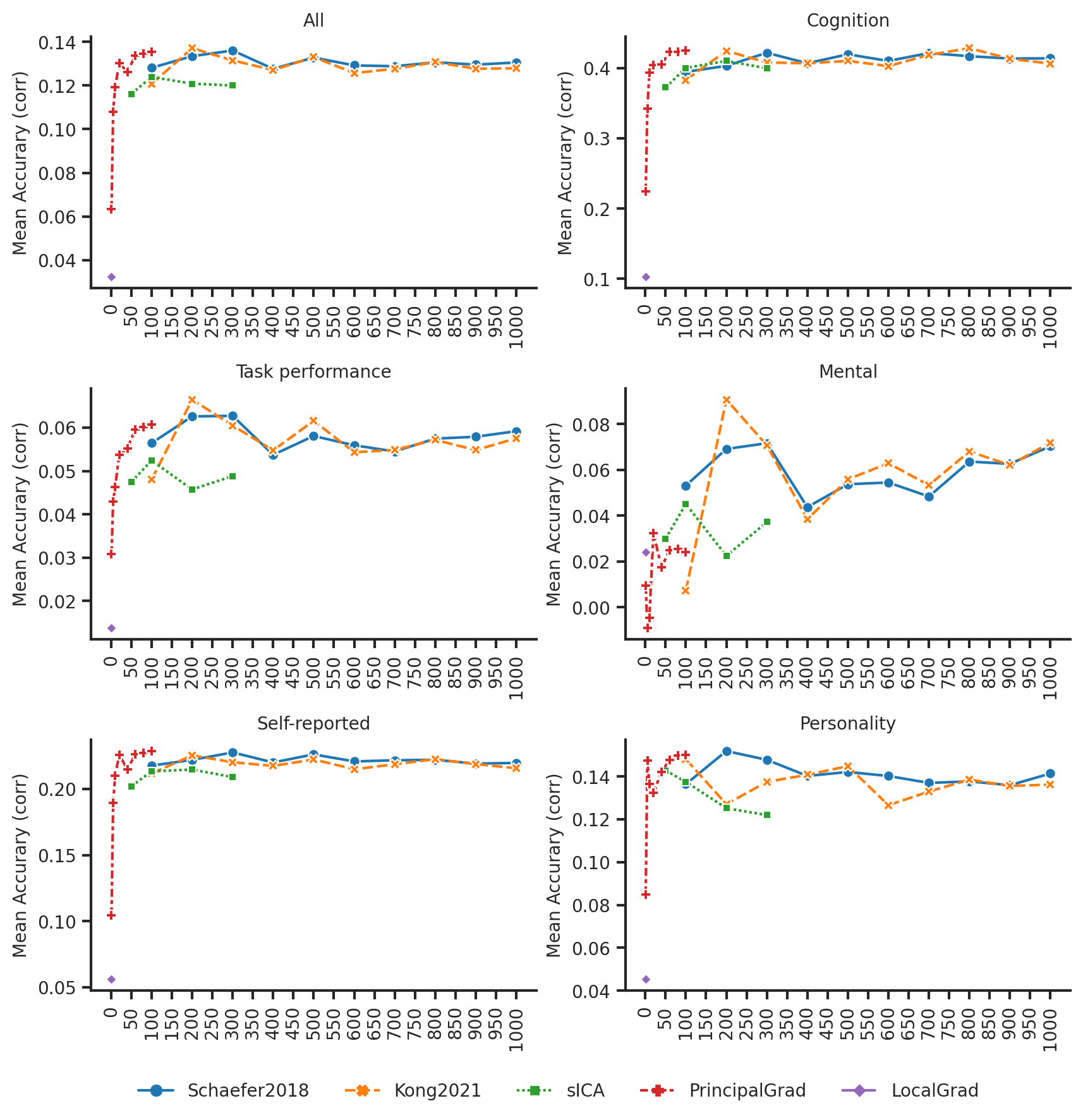


Figure S40. Prediction accuracies (Pearson’s correlation) vary across resolutions for gradient and parcellation approaches using KRR in the ABCD dataset.


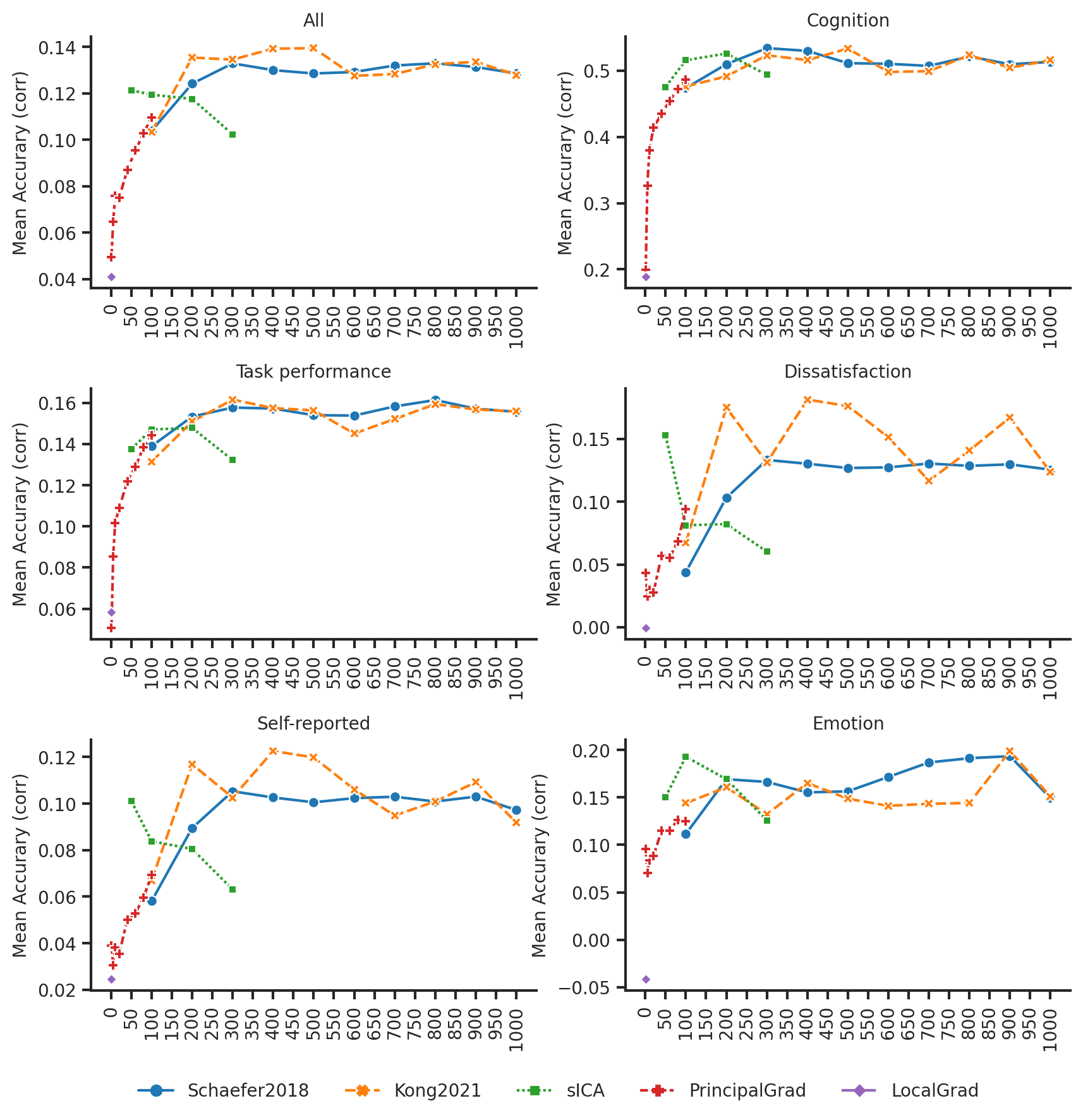


Figure S41. Prediction accuracies (Pearson’s correlation) vary across resolutions for gradient and parcellation approaches using LRR in the HCP dataset.


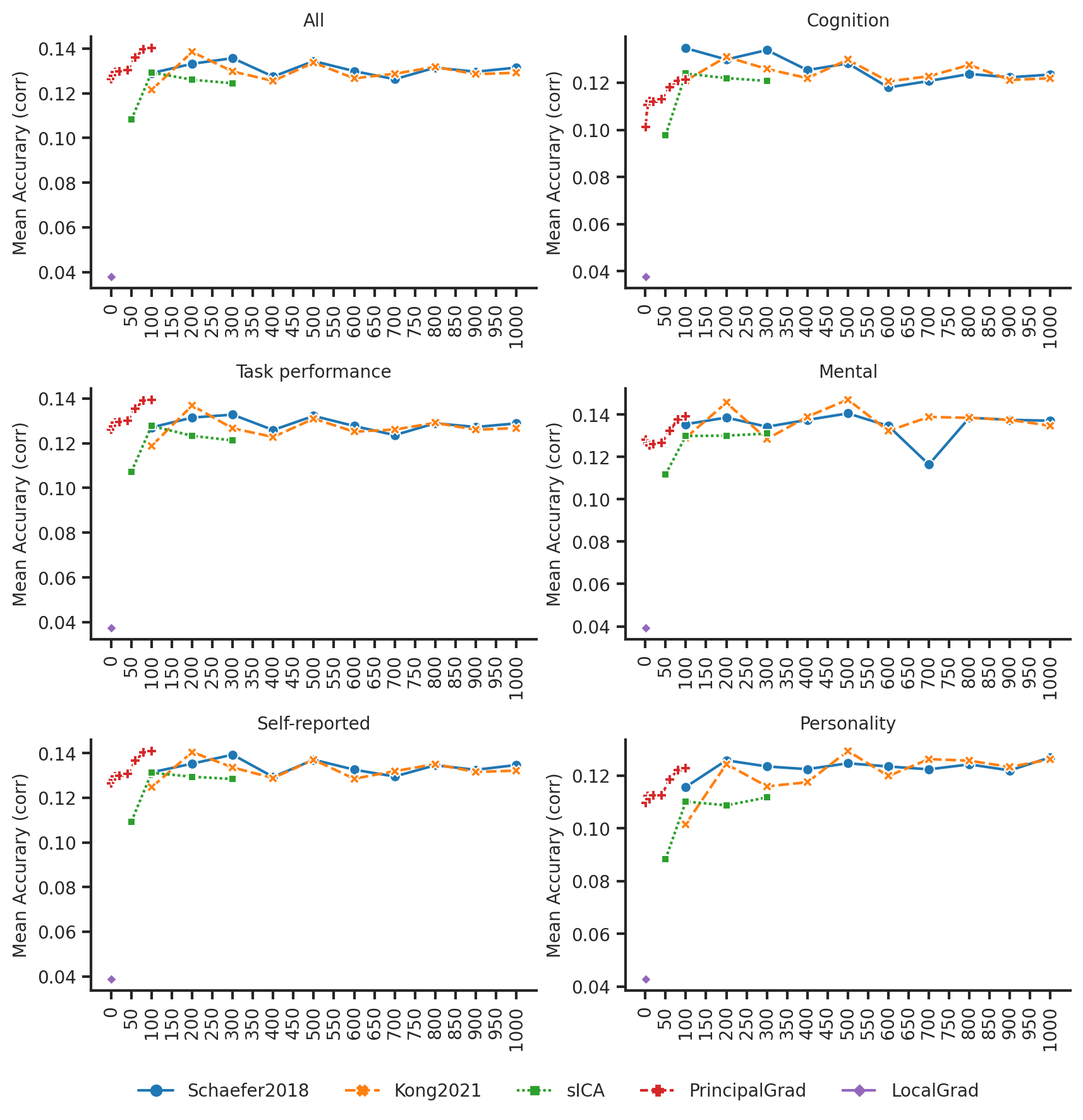


Figure S42. Prediction accuracies (Pearson’s correlation) vary across resolutions for gradient and parcellation approaches using LRR in the ABCD dataset.
